# Low salinity activates a virulence program in the generalist marine pathogen *Photobacterium damselae* subsp. *damselae*

**DOI:** 10.1128/msystems.01253-22

**Published:** 2023-06-08

**Authors:** Alba V. Barca, Ana Vences, Mateus S. Terceti, Ana do Vale, Carlos R. Osorio

**Affiliations:** 1 Departamento de Microbioloxía e Parasitoloxía, Instituto de Acuicultura, Universidade de Santiago de Compostela, Santiago de Compostela, Spain; 2 Fish Immunology and Vaccinology Group, IBMC-Instituto de Biologia Molecular e Celular, Universidade do Porto, Porto, Portugal; 3 i3S-Instituto de Investigação e Inovação em Saúde, Universidade do Porto, Porto, Portugal; University of California Merced, Merced, California, USA

**Keywords:** *Photobacterium damselae*, salinity, NaCl, transcriptome, secretome, virulence, hemolysin, arginine deiminase, damselysin

## Abstract

Facultative marine bacterial pathogens sense environmental signals so that the expression of virulence factors is upregulated on entry into hosts and downregulated during the free-living lifestyle in the environment. In this study, we utilized transcriptome sequencing to compare the transcriptional profiles of *Photobacterium damselae* subsp. *damselae*, a generalist pathogen that causes disease in diverse marine animals and fatal infections in humans at NaCl concentrations that mimic the free-living lifestyle or host internal milieu, respectively. We here show that NaCl concentration constitutes a major regulatory signal that shapes the transcriptome and uncover 1,808 differentially expressed genes (888 upregulated and 920 downregulated in response to low-salt conditions). Growth at 3% NaCl, a salinity that mimics the free-living lifestyle, upregulated genes involved in energy production, nitrogen metabolism, transport of compatible solutes, utilization of trehalose and fructose, and carbohydrate and amino acid metabolism with strong upregulation of the arginine deiminase system (ADS). In addition, we observed a marked increase in resistance to antibiotics at 3% NaCl. On the contrary, the low salinity conditions (1% NaCl) that mimic those encountered in the host triggered a virulence gene expression profile that maximized the production of the type 2 secretion system (T2SS)-dependent cytotoxins damselysin, phobalysin P, and a putative PirAB-like toxin, observations that were corroborated by the analysis of the secretome. Low salinity also upregulated the expression of iron-acquisition systems, efflux pumps, and other functions related to stress response and virulence. The results of this study greatly expand our knowledge of the salinity-responsive adaptations of a generalist and versatile marine pathogen.

**IMPORTANCE:**

Pathogenic *Vibrionaceae* species experience continuous shifts of NaCl concentration in their life cycles. However, the impact of salinity changes in gene regulation has been studied in a small number of *Vibrio* species. In this study, we analyzed the transcriptional response of *Photobacterium damselae* subsp. *damselae* (*Pdd*), a generalist and facultative pathogen, to changes in salinity, and demonstrate that growth at 1% NaCl in comparison to 3% NaCl triggers a virulence program of gene expression, with a major impact in the T2SS-dependent secretome. The decrease in NaCl concentration encountered by bacteria on entry into a host is proposed to constitute a regulatory signal that upregulates a genetic program involved in host invasion and tissue damage, nutrient scavenging (notably iron), and stress responses. This study will surely inspire new research on *Pdd* pathobiology, as well as on other important pathogens of the family *Vibrionaceae* and related taxa whose salinity regulons still await investigation.

## INTRODUCTION

Bacterial pathogens need to orchestrate the expression of virulence factors in a cell economy-driven fashion that guarantees the production of these factors only when and where they are actually needed. This is of special importance in generalist and facultative marine pathogens that thrive in a free-living lifestyle and also are capable of colonizing and infecting animals (1). In marine ecosystems, one of the main environmental signals that is highly informative of the transition from the free-living phase in seawater bodies to an infective stage is the abrupt drop of NaCl concentration encountered on entry into the internal milieu of an animal host.

The family *Vibrionaceae* comprises a genetically and metabolically diverse group of marine bacteria, and many species include animal and human pathogenic strains (2, 3). Most species enjoy a free-living existence in ocean water, attached to organic particles, and colonizing biotic and abiotic surfaces (4, 5). Thus, the association of pathogenic *Vibrios* with animals can be considered an optional part of their life cycle, and only a very reduced number of species might be viewed as obligate pathogens. Though pathogenic *Vibrionaceae* species experience continuous shifts of NaCl concentration in their life cycles, very few species of this family have been the subject of global transcriptomic studies aimed at investigating the role of salinity in gene expression modulation. These include *Vibrio cholerae* (6, 7), *Vibrio parahaemolyticus* (8 - 10), *Vibrio brasiliensis* (11), and *Vibrio fujianensis* (12). However, some of these studies focused on the study of salt stress and employed NaCl conditions that do not mimic those encountered in nature (12). Other studies compared conditions that differed in NaCl concentrations but also in additional variables, as is the case of a study with *Vibrio vulnificus* grown in human serum versus seawater (13).

*Photobacterium damselae* subsp. *damselae* (hereafter *Pdd*), formerly known as *Vibrio damsela*, is a member of the *Vibrionaceae* that has been isolated in marine ecosystems from diverse sources such as coastal ocean waters (14), sediment (15), shellfish (16), marine bird droppings (17), digestive tract of whales (18), fish and shrimp intestines (19, 20), and internal organs of apparently healthy fish (21, 22). It is also a strong histamine-producing bacterium commonly isolated from the retailed fish (23). In addition, *Pdd* is a highly versatile and virulent generalist pathogen of increasing financial concern that causes primary disease in crustaceans and in countless species of fish, wild as well as cultivated in aquaculture facilities in different parts of the globe (24, 25). Of note, *Pdd* is also an opportunistic human pathogen that can cause wound-associated infections on contact with the marine milieu and fatal necrotizing fasciitis (26).

The highly virulent strains of *Pdd* cause massive tissue damage in the infected hosts due to the plasmid (pPHDD1)-encoded cytotoxins damselysin (Dly) and phobalysin P (PhlyP), and the chromosome-encoded phobalysin C (PhlyC), which are secreted in very high amounts by the type 2 secretion system (T2SS) (27). Dly is a phospholipase D active against sphingomyelin, whereas PhlyP and PhlyC are pore-forming toxins. Additional demonstrated virulence factors of *Pdd* include a polysaccharide capsule that contributes to *Pdd* resistance to host defense mechanisms (28).

The biological and environmental signals that regulate gene expression in *Pdd* and, specifically, virulence factors are scarcely understood. Expression of the cytotoxins and biogenesis of the polysaccharide capsule are positively regulated by the two-component system RstAB (27 - 29), homologous to the *V. cholerae* CarSR (30), but the specific signal that triggers the activation of the RstAB system remains unknown. Previous studies have reported that iron limitation upregulates the expression of genes encoding Dly, PhlyP, and PhlyC cytotoxins (31, 32). Also, NaCl levels were found to modulate the transcriptional activity of the promoters of the three aforementioned cytotoxins (31), as well as phospholipase and hemolytic activities (33), but no further insights have been taken to study the role of salinity in *Pdd* gene regulation. In this study, we use RNA sequencing (RNA-seq) to analyze the transcriptional profiles of the highly virulent *Pdd* strain RM-71 grown at average seawater salinity (3% NaCl) and at the salinity of the internal milieu of vertebrate hosts (1% NaCl), respectively. Our results reveal that a shift from high to low salinity triggers a virulence gene expression profile with a strong impact in the T2SS-dependent secretome, including the upregulation of cytotoxins, iron-acquisition systems, and other functions potentially involved in pathogenicity.

## MATERIALS AND METHODS

### Standard culture conditions

*Pdd* strains were routinely grown at 25°C on tryptic soy agar (TSA) or tryptic soy broth (TSB) and in M9 minimal medium (34) supplemented with 0.2% casamino acids (Difco) (CM9) and 0.5% glucose. NaCl was adjusted to final concentrations of 1% (TSA-1, TSB-1, CM9-1) or 3% (TSA-3, TSB-3, CM9-3), as necessary. When necessary, antibiotics were supplied at the following final concentrations: kanamycin (Km) at 50 µg/mL and rifampicin (Rif) at 20 µg/mL.

### RNA extraction and RNA-seq

The highly virulent *Pdd* strain RM-71 selected for the present study carries the virulence plasmid pPHDD1 and was isolated from a diseased turbot in 1988 during an outbreak in Galicia (northwest Spain) (35). For RNA-seq, three independent precultures of RM-71 for each salinity condition (1% NaCl and 3% NaCl) in TSB were grown until they reached an optical density at 600 (OD_600_) of 0.3. Then, each preculture was diluted (1:100) and grown in 10-mL TSB until an OD_600_ of 0.55. At this point, cultures were instantly treated with RNAprotect Bacteria Reagent (Qiagen) for RNA stabilization. Pelleted cells were resuspended in TE buffer (30 mM Tris-HCl, 1 mM EDTA, pH 8.0) with 10-µL lysozyme (15 mg/mL) (Sigma-Aldrich) and 15-µL Proteinase K (20 mg/mL) (Qiagen). The RNeasy Mini Kit (Qiagen) was used for RNA extraction and on-column digestion of DNA with DNase I was performed using the RNase-free DNase kit (Qiagen). The integrity and the amount of the total RNA were evaluated using a Bioanalyzer 2100 (RNA 6000 Nano chip assay) and a Qubit 3.0 (Quant-It dsRNA BR Assay). rRNA depletion was performed with the Ribo-Zero rRNA Removal Kit (Gram-negative bacteria) (Illumina), and cDNA libraries were generated using the TruSeq RNA kit in accordance with Illumina’s instructions. First, rRNA-depleted RNA was chemically fragmented before being subjected to reverse transcription for cDNA synthesis. A reparative process was performed by adding a single “A” base to the 3′ end of cDNA fragments following adapters’ ligation. Finally, cleaned products were enriched by PCR to generate the double-stranded cDNA library that was sequenced on an Illumina HiSeq 2500 sequencer.

### RNA-seq data analysis

RNA-seq raw reads were analyzed with FastQC program as previously described (36) and mapped against the genome of RM-71 (RefSeq assembly accession no.: GCF_001708035.2) using the Bowtie2 v2.2.6 algorithm (37). Poor-quality readings were eliminated using PicardTools (http://picard.sourceforge.net). Several quality steps were performed to evaluate sequencing and mapping processes. Distribution of GC content and duplicate proportion of mappable readings were evaluated. Genetic quantification was performed by the HTSeq software (0.6.1 version) (38). A correlation and distance study between samples from the same condition was carried out to evaluate them as biological replicates. For this, the transcriptome normalized by the size of the library was analyzed with statistics program R. The analysis of the differential expression was performed using DESeq2 method (1.18.1 version) (39), and a differential negative binomial distribution was applied to determine statistical significance. A Python script developed at Sistemas Genómicos (Valencia, Spain) was employed to generate a data matrix with the counts obtained for each sample (each of the three replicates at each of the two salinities). Genes were considered as differentially expressed when fold change (FC) values were lower than −1.5 or higher than 1.5 and a *P*-value adjusted by false discovery rate (FDR) ≤ 0.05. Differentially expressed genes (DEGs) were mapped against Uniprot, COG (Cluster of Orthologous Groups), GO (Gene Ontology) and KEGG (Kyoto Encyclopaedia of Genes and Genomes) databases and analyzed using the hyper-geometric test. An FDR-adjusted *P*-value of 0.05 was used to determine a functional category as statistically significant or over-represented (40).

### Growth experiments

For growth assays, an inoculum of *Pdd* RM-71 strain adjusted to an OD_600_ of 0.3 was diluted (1:100) in 100 µL of the tested media in a 96-well plate. Bacterial cultures were incubated at 25°C under shaking conditions, and growth was monitored for 24–48 hours using the spectrophotometer Epoch2 microplate reader (BioTek). The experiments were independently performed twice, and at least two replicates per condition were included. Growth media were prepared according to the aim of the experiments as detailed below.

- **Growth in seawater microcosms**. To evaluate *Pdd* growth in marine-like microcosms, coastal seawater samples were collected at Lodeiro beach, Rianxo, Galicia (northwest Spain). One sample consisting of plain seawater without macroscopically visible particulate material was sterilized in an autoclave and named as NSW (natural seawater). A second sample of 100-mL seawater was supplemented with 30 g (wet weight) of macroalgae (*Ulva lactuca*) occurring in the surroundings of the same collection site, and the mix was mechanically ground and then boiled at 95°C for 1 minute and labeled as ESW (eutrophicated seawater). The two types of samples were supplemented with 0.2% casamino acids (Difco) when necessary. For growth experiments, three replicates per condition were prepared using an inoculum of *Pdd* RM-71 adjusted to an OD_600_ of 0.3 and diluted (1:100) in 100 µL of NSW or ESW in a 96-well plate. Bacterial growth was monitored for 24 or 48 hours using the spectrophotometer Epoch2 microplate reader (BioTek) under shaking conditions.

- **Carbohydrate-supplemented minimal medium**. *Pdd* RM-71 strain was tested for the ability to ferment the carbohydrates fructose and trehalose under different NaCl concentrations. For this purpose, OF (Oxidation/Fermentation) basal medium (Difco) was supplemented with agar (15 g/L) and NaCl to a final concentration of 1% or 3%. Fructose and trehalose (Sigma-Aldrich) were added individually to a final concentration of 0.5%, and plates were streaked with RM-71 strain from a freshly grown TSA-1 plate. Plates were incubated at 25°C and monitored for 3 days. The ability to ferment the sugars was indicated by a color change from green to acidic yellow. For growth experiments, trehalose-minimal medium and fructose-minimal medium were prepared by supplementing CM9 minimal medium with 0.5% of each carbohydrate. NaCl concentrations were adjusted to final concentrations of 1% and 3%, as necessary. Growth in CM9-minimal medium with 0.5% glucose at both NaCl concentrations was included in the experiment as a control.

- **Growth with antibiotics under different NaCl concentrations**. To assess the impact of NaCl concentration on growth in the presence of antibiotics, *Pdd* RM-71 was grown in TSB-1 and TSB-3 supplemented with kanamycin (50 µg/mL), ampicillin (100 µg/mL), and vancomycin (25 µg/mL). After 40 hours, cells grown in TSB-3 were passaged (1:100) to fresh antibiotic-supplemented TSB medium at both NaCl conditions in a 96-well plate and growth was monitored for 40 hours.

- **Antimicrobial activity of piscidin peptides against *Pdd.*** Piscidin peptide 1 (PP1) was designed and produced as described by Barroso et al. (41). PP1 at 15 µM (previously determined minimum inhibitory concentration) was serially diluted (1:2) up to a concentration of 0.12 µM and incubated with *Pdd* RM-71 (10^8^ CFU/mL) on TSB-1 and TSB-3 in a final volume of 100 µL in 96-well plates. Wells without bacteria and wells with no added peptide were used as blanks and controls, respectively.

### Hemolysis and phospholipase assays

Hemolysis and phospholipase activities were evaluated on TSA plates supplemented with 5% sheep blood agar (Thermo Scientific) and 3% egg yolk extract (Oxoid), respectively. To determine the impact of NaCl concentration in the phenotypical observation of these two activities, NaCl was added to a final concentration of 1% or 3% during plate preparation. RM-71 cultures were grown in TSB-1 and TSB-3 up to an OD_600_ of 1.2, and 2 µL of the suspension were spotted on each assay plate and incubated at 25°C for 24–48 hours. The hemolytic and phospholipase activities were calculated by dividing the halo diameter by the colony diameter. A Student’s *t*-test was used to determine statistical significance.

### Motility assays

Motility was determined using the swim-migration assay (42). For this, 4 µL of cultures grown on TSB-1 or TSB-3 up to an OD_600_ of 0.2 were vertically inoculated into a semisolid TSA plate, containing 0.25% agar, 10 mM of L-arginine (Sigma-Aldrich), and either 1% or 3% NaCl. Plates were incubated at 25°C under aerobic and anaerobic conditions for 24 hours and motility haloes were measured. An AnaeroJar containing AnaeroGen sachets (Thermo Fisher Scientific) was used to generate the anaerobic conditions, and resazurin strips (Thermo Fisher Scientific) were used to confirm the absence of oxygen during incubation.

### Arginine decarboxylase/dihydrolase test

Arginine Moeller’s medium was used to test arginine decarboxylase/dihydrolase activity by dissolving 10 g/L of Decarboxylase Base Moeller (Difco), 10 g/L of L-arginine (Sigma-Aldrich), and supplemented with 1% or 3% NaCl. Tubes were inoculated with bacteria precultured in TSB-1 or TSB-3 medium to an OD_600_ of 1.4 and covered with 1 mL of sterile mineral oil. A non-inoculated tube was included as a negative control. Tubes were incubated at 25°C for 48 hours and monitored every 12 hours for changes.

### Acid survival assays under different NaCl conditions

To investigate the effect of salinity on *Pdd* sensitivity to acid, growth kinetics at various pH levels and survival assays were determined under different NaCl concentrations. For growth assays, three replicates of RM-71 adjusted to an OD_600_ of 0.3 were inoculated (1:100) in 100 µL of TSB-1 and TSB-3 adjusted to pH 7, 6, 5, and 4. Growth was monitored for 48 hours using the spectrophotometer Epoch2 microplate reader (BioTek) under constant stirring. To determine bacterial viability on acid exposure, overnight cultures of RM-71 and ∆*arcA* strains in 5-mL of TSB-1 (pH 7) were diluted in fresh TSB-1 and TSB-3 and grown to an OD_600_ of 0.3. Bacterial cells were harvested (4,000 × *g* for 5 minutes) and suspended in 1 mL of acidified TSB-1 and TSB-3 (pH 4). Then, 1:100 dilutions were inoculated in TSB-1 and TSB-3 (pH 4.0), with and without supplemental L-arginine (10 mM) (Sigma-Aldrich), in 96-well plates. For colony counts, 5-µL aliquots of 10-fold dilutions at 0 and 16 hours were drop-plated in TSA-1. Plates were incubated at 25°C for 24 hours, and the number of viable cells (CFU/mL) was determined for each time point.

### Scanning and transmission electron microscopy (SEM and TEM)

For SEM, exponentially growing cultures of *Pdd* RM-71 in TSB-1 and TSB-3 (OD_600_ of 0.55) were pelleted down by centrifugation (4,000 × *g*, 5 minutes, 4°C). Cells were fixed as previously described (27), sputter-coated with iridium, and imaged using an Ultra Plus ZEISS scanning electron microscope. For polysaccharide capsule visualization, sample processing and TEM analyses were conducted as previously described (28). Images were digitally recorded using a CCD digital camera Orius 1100 W (Gatan). Cell length, cell width, and capsule thickness at both salinities were determined by measuring 30 cells with the Fiji software (ImageJ version 1.51n) (43). Capsule thickness for each cell was calculated as the average of six measurements at different points. An unpaired *t*-test was used to determine statistical significance.

### Allelic exchange mutagenesis

Non-polar deletion mutants for δ-endotoxin gene *pirB* (A0J47_RS13275) and arginine deiminase *arcA* (A0J47_RS14010) were constructed by allelic exchange mutagenesis. Primers used for the construction and screening of mutants are listed in Table S3. In brief, 2-kb DNA fragments upstream and downstream of each gene were obtained by amplification with Hi-Fidelity Kapa Taq (*Kapa*) and subsequently ligated, resulting in an in-frame deletion of >90% of the coding sequence for each target gene. For allelic exchange, the suicide vector pNidKan (Km^R^), bearing the sucrose sensitivity gene *sacB* and the pir-dependent R6K *ori,* was used as previously described (28). To select the first event of recombination, 100-µL serial dilutions of transconjugants were seeded on thiosulfate-citrate-bile salts-sucrose (TCBS) agar supplemented with kanamycin. Colonies that were resistant to kanamycin were seeded on TSA-1 plates supplemented with sucrose (15% [wt/vol]) to select for a second recombination event. As a control to study whether PirAB toxin has an impact on the virulence of *Pdd* for fish, we needed to construct a *Pdd* RM-71 mutant defective for four previously characterized toxins Dly, PhlyP, PhlyC, and PlpV (31, 44). Such a tetra-mutant (dubbed here AVL442) lacks these four toxins but maintains intact *pirA* and *pirB* genes. This tetra-mutant was constructed by deleting *plpV* as previously described (44) on the genetic background of the previously constructed triple (Dly, PhlyP, PhlyC) mutant AR89 (31). All gene deletions were confirmed by PCR, and the DNA regions involved in recombination were sequenced to ensure that no point mutations were generated during the process.

### SDS-PAGE analysis of culture supernatants

To analyze the effect of NaCl changes on the abundance of secreted proteins, the extracellular products (ECPs) from three replicates of *Pdd* RM-71 cultures grown in TSB-1 or TSB-3 were collected. In a first attempt, to be able to correlate the protein profiles with the transcriptomic results, we obtained ECPs from cultures obtained in the same conditions used for RNA-seq analysis (OD_600_ of 0.55). However, due to the low protein abundance in these early exponential supernatants, for the analysis of the secretome we used cultures grown to stationary phase (OD_600_: 1.7). Bacterial suspensions were centrifuged (13,000 × *g*, 5 minutes, 4°C), and supernatants were filtered through 0.22-µm-pore size filters (Schleicher & Schuell, Dassel, Germany). Protein precipitation of cell-free supernatants was performed as previously described (27), and precipitated proteins were subjected to SDS-PAGE in 14% polyacrylamide gels using the Laemmli discontinuous buffer system (45). Coomassie Brilliant Blue R250 was used for protein staining.

### Protein quantification by densitometry

SDS-PAGE gel images acquired in a GS-900 calibrated densitometer (Bio-Rad) were analyzed using the Image Lab Software version 6.0.1 (Bio-Rad). Bands were automatically detected by the software by selecting the high-sensitivity setting. Rolling disk values were set for the lane with less intensity, applied to all lanes, and adjusted to determine the background-subtracted intensity for each band. Normalization between lanes was determined using the sialidase band as a housekeeping secreted protein (HKP) given its stable expression under the experimental conditions tested. For this, the lane normalization factor (LNF) was calculated for each gel by dividing the HPK signal of each lane by the HPK value with the highest intensity. Then, normalized band intensity was determined for each target protein by dividing its signal value by the corresponding LNF. Mean of normalized band intensities of three replicates from independent cultures for each condition (1% NaCl and 3% NaCl) over three gels was calculated for each protein. An unpaired, two-tailed Student’s *t-*test was used for the statistical analysis of densitometric data using the GraphPad Prism 9 Software.

### Fish virulence assays

For experimental infection assays on turbot, groups of 10 fish (6 ± 1.2 g) were acclimated at 18°C for 1 week before the infection assay. Virulence challenges were conducted by intraperitoneal injection of 0.1 mL of bacterial suspensions in 0.85% NaCl solution at a dose of 4 × 10^5^ CFU/fish per all the tested strains. A negative control group of 10 fish was inoculated with 0.1 mL of sterile 0.85% NaCl. Fish mortality was recorded for 7 days. Reisolation on TSA-1 and TCBS and identification of bacteria from the kidney of dead fish was performed by PCR with *ureD* primers (46). The protocols of animal experimentation used in this study have been reviewed and approved by the Animal Ethics Committee of the Universidade de Santiago de Compostela.

## RESULTS AND DISCUSSION

### General overview of the transcriptional profile of *Pdd* RM-71 under different conditions of salinity

**Fig 1:**
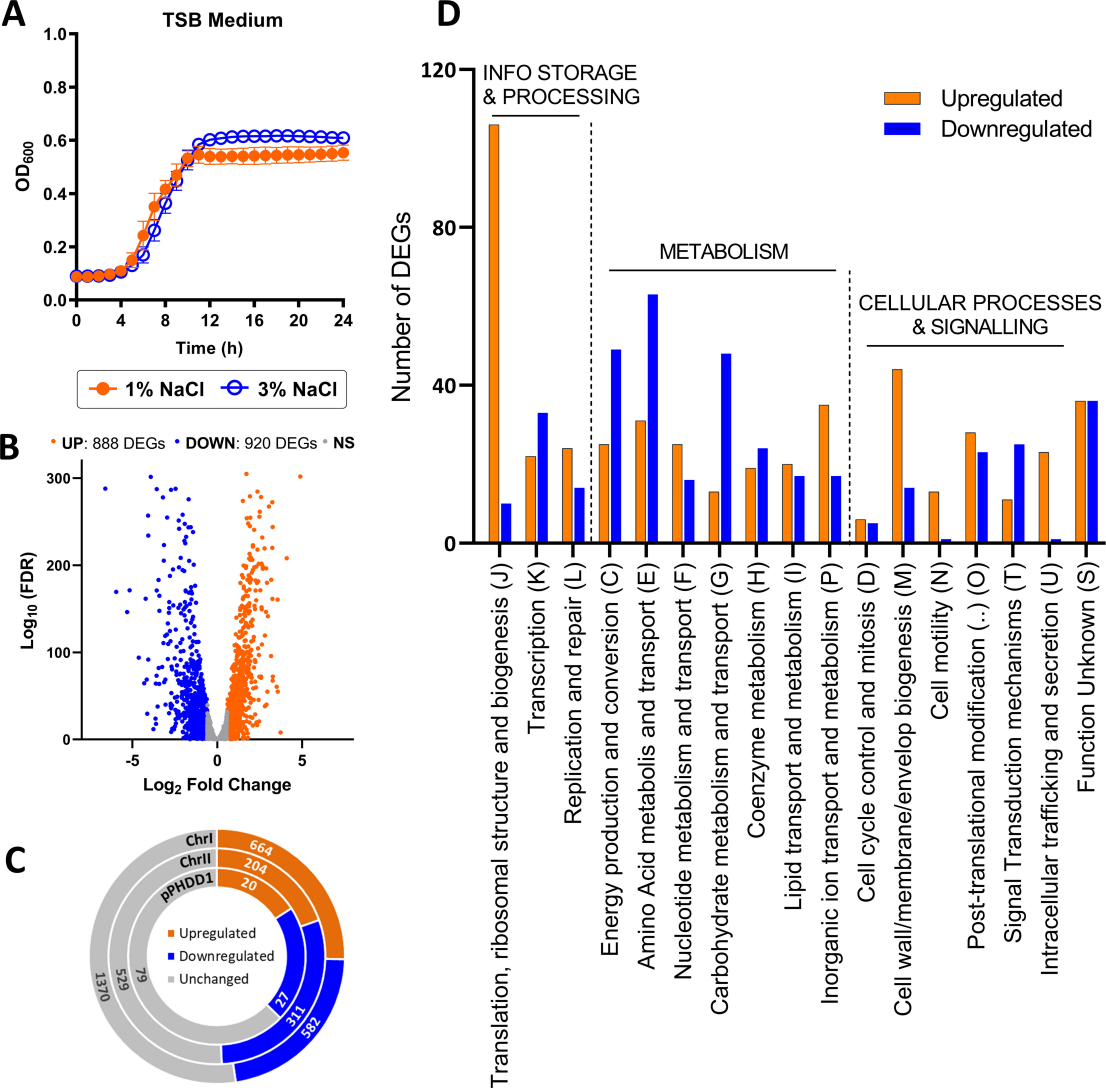
(**A**) Growth of *Pdd* RM-71 cultured in TSB medium at 1% and 3% NaCl. Data are presented as mean ± SD from three biological replicates and three independent experiments. (**B**) Volcano plot showing statistical significance of fold change (FC) values. Genes with FDR adjusted *P*-values ≤ 0.05 and Log_2_ FC values ≥ 0.59 and ≤ –0.59 were considered to be upregulated DEGs (orange dots) or downregulated DEGs (blue dots) at 1% NaCl. (**C**) Distribution of the number of upregulated (orange), downregulated (blue), and unchanged (gray) genes within the genome of strain RM-71 at 1% NaCl versus 3% NaCl. Distribution of DEGs is balanced in both chromosomes as well as in the virulence plasmid pPHDD1. (**D**) Functional classification of upregulated (orange) and downregulated (blue) DEGs at 1% NaCl versus 3% NaCl based on COG (Cluster of Orthologous Groups) database.

*Pdd* requires the addition of NaCl for growth and is capable of growing at 1%, 3%, 5%, and 6% NaCl (47). To assess the impact of NaCl concentration on *Pdd* growth dynamics, precultures of the highly virulent strain RM-71 prepared at 1% NaCl in TSB medium were used as starters (1:100 dilution) of new cultures at two different salinities, 1% or 3% NaCl. *Pdd* achieved a slightly higher optical density at 3% NaCl at the end of the exponential phase (Fig. 1A).

To investigate the transcriptional response of *Pdd* to NaCl changes, triplicates of early exponential RM-71 cultures grown in TSB at 3% NaCl and 1% NaCl were used to purify RNA and to generate six cDNA libraries that were subjected to Illumina sequencing. An average of 54.27 million raw reads were generated and mapped against the complete genome of *Pdd* RM-71. Principal component analysis demonstrated a clear distinction between the two sample groups (1% NaCl and 3% NaCl) (Fig. S1). Detailed information on the RNA-seq data for each replicate is described in Table S1. To visualize the changes in gene expression that might be elicited in *Pdd* on entry into an animal host, growth at 3% NaCl was established as the control condition in comparison to growth at 1% NaCl. The comparative analysis of the transcriptomic profiles identified 1,808 DEGs: 888 upregulated (FC > 1.5) and 920 downregulated (FC < −1.5) at 1% NaCl compared to 3% NaCl (Fig. 1B). The complete list of DEGs at each salinity condition is summarized in Table S2. The number of upregulated and downregulated genes was balanced in the two chromosomes, as well as in the virulence plasmid pPHDD1 (Fig. 1C).

The proteins encoded by the DEGs were mapped to COG, KEGG, and GO databases. Interestingly, translation (J) was the most abundant functional category assigned by the COG database within the low salt-upregulated profile (Fig. 1D). In addition, genes upregulated at 1% NaCl were mostly assigned to functional categories involved in inorganic ion metabolism and transport (P), membrane biogenesis (M), cell motility (N), and intracellular secretion and trafficking (U). Conversely, categories related to general metabolism, such as energy production and conversion (C), amino acid and carbohydrate metabolism and transport (E and G, respectively) were the most represented among genes upregulated at 3% NaCl. Figures showing the top 15 enriched KEGG pathways and GO terms involved in biological processes are presented in Fig. S2.

**Fig 2:**
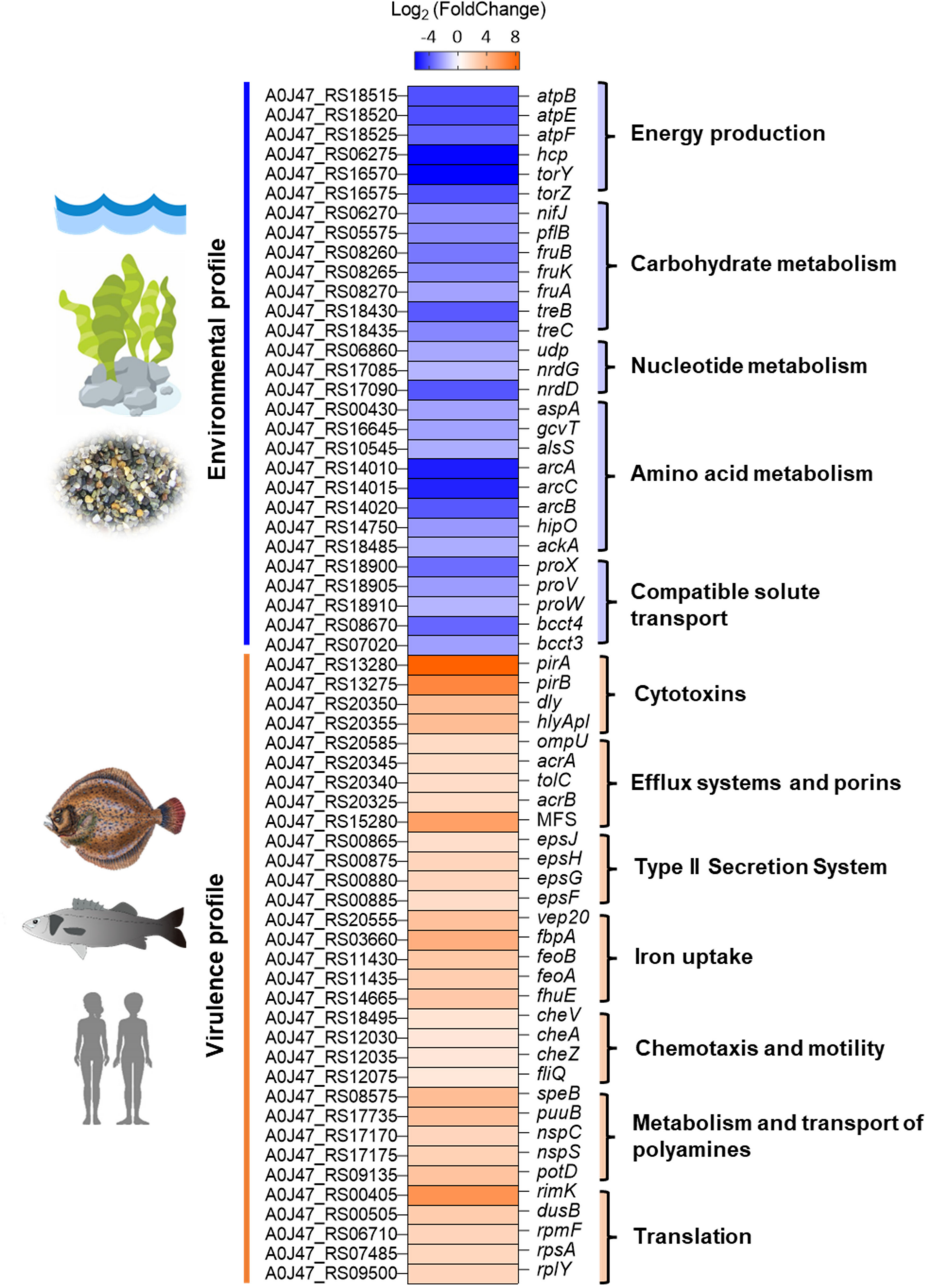
Heat map view showing the general transcriptional response of *Pdd* at 1% NaCl (upregulation of a virulence profile) compared to 3% NaCl (upregulation of an environmental profile). Differential expression values are given as Log_2_FC-based color scale. Orange segments represent induced expression (positive FC), whereas blue segments represent downregulation (negative FC).

**Fig 3:**
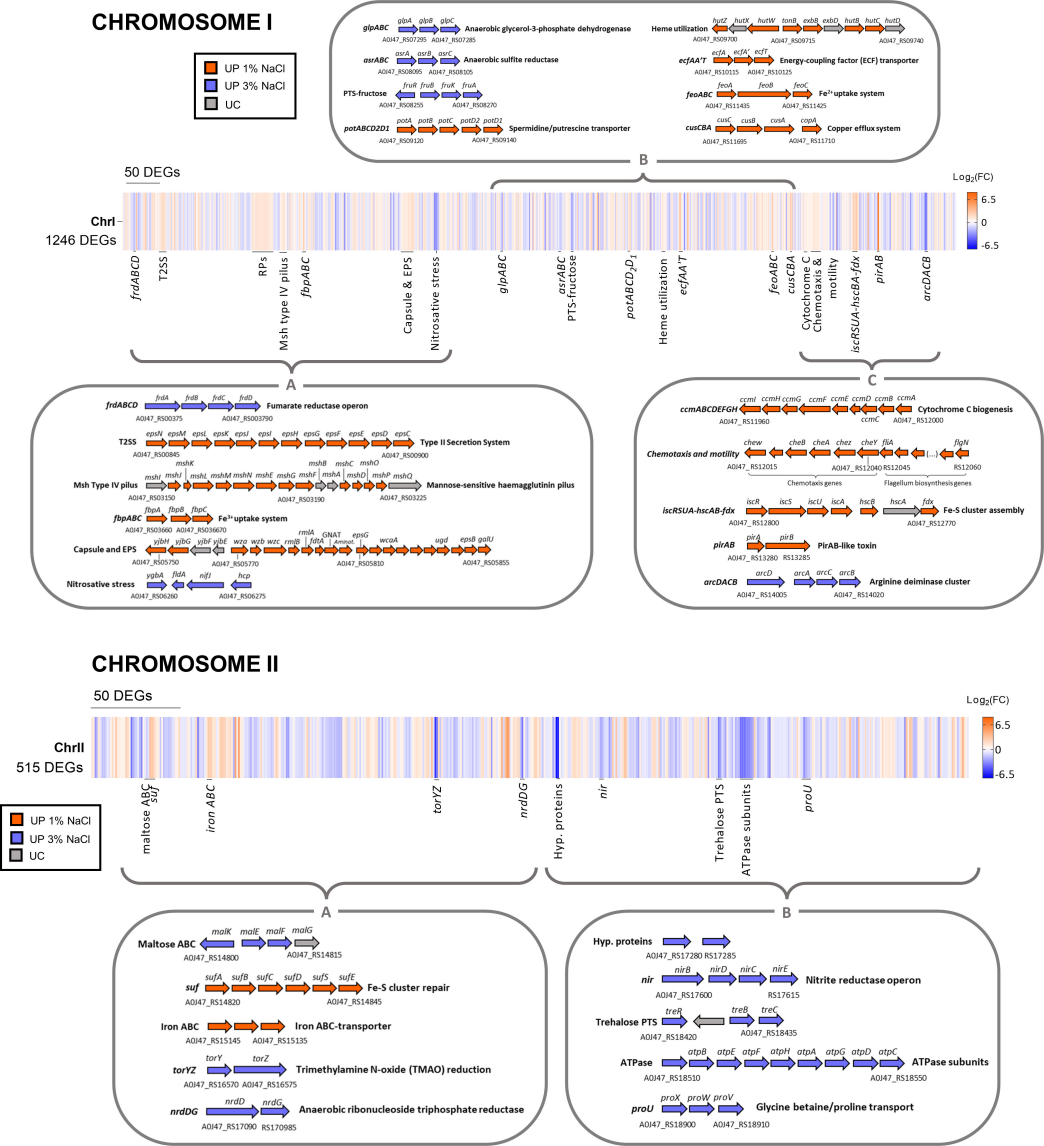
Mapping of differentially expressed genes (DEGs) in response to NaCl changes within chromosome I and chromosome II of *Pdd* RM-71 genome. Expression values are depicted in a color-based scale as Log_2_fold change (FC). In the detailed view, DEGs are shown in orange (upregulated at 1% NaCl), blue (upregulated at 3% NaCl), and gray (unchanged) and annotated with the corresponding National Center for Biotechnology Information (NCBI) Locus Tag of RM-71 genome (GCF_001708035.2). Operon-predicted functions are shown.

As a general overview, the comparative study of the transcriptomes revealed the upregulation of genes involved in energy metabolism, biosynthesis of amino acids, ADS, and uptake of compatible solutes at 3% NaCl (Table 1), whereas growth at 1% NaCl upregulated genes encoding cytotoxins and other proteins secreted via the T2SS, iron uptake systems, and other virulence factors (Table 2). Therefore, we propose that growth at 3% NaCl upregulates an “environmental profile” in *Pdd*, whereas a shift to 1% NaCl upregulates a “virulence profile” (Fig. 2). Study of DEG distribution along the two chromosomes of the *Pdd* RM-71 genome revealed that some of the top DEGs mapped to multigene operons (Fig. 3). The most relevant results obtained from the detailed study of the transcriptomes are discussed below.

**TABLE 1:** List of selected DEGs significantlyater samples were collected at L upregulated at 3% NaCl

Locus_tag	Product/function	FC	Log_2_FC	*P*-value	Location ^*a*^
***Energy metabolism***					
A0J47_RS18510	Hypothetical protein	–19.83	–4.31	4.52E-66	ChrII
A0J47_RS18515	F_0_F_1_ ATP synthase subunit a	–16.75	–4.07	1.31E-93	ChrII
A0J47_RS18520	F_0_F_1_ ATPase subunit c	–17.46	–4.13	7.67E-31	ChrII
A0J47_RS18525	F_0_F_1_ ATPase subunit b	–12.28	–3.62	1.12E-65	ChrII
A0J47_RS18540	F_0_F_1_ ATPase subunit gamma	–10.65	–3.41	4.22E-82	ChrII
A0J47_RS00375	Fumarate reductase flavoprotein subunit FrdA	–3.95	–1.98	4.53E-76	ChrI
A0J47_RS16570	Cytochrome c-type subunit TorY	–62.18	–5.96	1.56E-17	ChrII
A0J47_RS16575	Putative molybdoenzyme reductase TorZ	–16.80	–4.07	1.86E-23	ChrII
A0J47_RS08095	Anaerobic sulfite reductase subunit AsrA	–2.20	–1.14	2.52E-07	ChrI
***Nitrogen metabolism and nitrosative stress response***					
A0J47_RS06275	Hydroxylamine reductase	–58.34	–5.87	0	ChrI
A0J47_RS17605	Nitrite reductase (NADH) small subunit	–6.58	–2.72	1.61E-09	ChrII
A0J47_RS06260	Nitrous oxide-stimulated promoter family protein	–18.74	–4.23	2.83E-162	ChrI
A0J47_RS00170	Nitric oxide-sensing transcriptional repressor NsrR	–1.60	–0.68	5.44E-30	ChrI
***Carbohydrate metabolism***					
***Pyruvate metabolism and acetate production***					
A0J47_RS00915	Phosphoenolpyruvate carboxykinase PckA	–2.13	–1.09	2.41E-61	ChrI
A0J47_RS06270	Pyruvate-ferredoxin/flavodoxin oxidoreductase NifJ	–6.59	–2.72	4.31E-37	ChrI
A0J47_RS07640	Alcohol dehydrogenase	–2.69	–1.43	1.88E-41	ChrI
A0J47_RS13735	Pyruvate-formate lyase GrcA	–27.94	–4.80	0	ChrI
A0J47_RS05575	Formate C-acetyltransferase PflB	–6.60	–2.72	3.94E-287	ChrI
A0J47_RS05580	Formate efflux transporter FocA	–6.10	–2.71	6.74E-196	ChrI
A0J47_RS18485	Acetate kinase AckA	–3.80	–1.93	3.18E-84	ChrII
***Fructose and mannose metabolism***					
A0J47_RS05660	Mannose-6-phosphate isomerase ManA	–1.58	–0.66	2.77E-41	ChrI
A0J47_RS05670	PTS system, fructose-specific IIA component	–4.47	–2.16	9.64E-21	ChrI
A0J47_RS08255	Fructose operon transcriptional repressor FruR	–3.74	–1.90	1.58E-67	ChrI
A0J47_RS08260	PTS system, fructose-specific IIA component FruB	–8.93	–3.16	1.91E-20	ChrI
A0J47_RS08265	1-phosphofructokinase FruK	–6.91	–2.79	1.44E-12	ChrI
A0J47_RS08270	PTS system, fructose-specific IIB/C component FruA	–4.48	–2.16	2.19E-77	ChrI
***Starch and glucose metabolism***					
A0J47_RS15340	Glycogen phosphorylase	–3.14	–1.65	2.10E-67	ChrII
A0J47_RS18420	Trehalose operon repressor TreR	–2.13	–1.09	3.69E-51	ChrII
A0J47_RS18430	PTS system, trehalose-specific IIBC component PTS system TreB	–14.92	–3.90	0	ChrII
A0J47_RS18435	Trehalose-6-phosphate hydrolase TreC	–7.00	–2.81	1.83E-30	ChrII
***Amino sugar and nucleotide metabolism***					
A0J47_RS17085	Anaerobic ribonucleoside-triphosphate reductase activating protein NrdG	–3.39	–1.76	8.82E-58	ChrII
A0J47_RS17090	Anaerobic Ribonucleoside-triphosphate reductase (thioredoxin) NrdD	–15.70	–3.97	0	ChrII
A0J47_RS06860	Uridine phosphorylase Udp	–4.12	–2.04	4.58E-195	ChrI
A0J47_RS11155	N-acetylneuraminate lyase NanA	–4.65	–2.22	1.38E-75	ChrI
***Amino acid metabolism***					
A0J47_RS14010	Arginine deiminase ArcA	–39.70	–5.31	6.85E-147	ChrI
A0J47_RS14015	Carbamate kinase ArcC	–35.99	–5.17	7.93E-172	ChrI
A0J47_RS14020	Ornithine carbamoyltransferase ArcB	–15.11	–3.92	3.70E-302	ChrI
A0J47_RS14005	Arginine/ornithine antiporter ArcD	–2.20	–3.12	0	ChrI
A0J47_RS02700	Arginine N-succinyltransferase AstA	–2.28	–1.19	1.46E-60	ChrI
A0J47_RS02705	Succinylglutamic semialdehyde dehydrogenase AstD	–2.35	–1.23	4.96E-40	ChrI
A0J47_RS01975	Arginine decarboxylase AdiA	–2.20	–1.14	8.73E-29	ChrI
A0J47_RS00430	Aspartate ammonia-lyase AspA	–4.51	–2.17	1.73E-111	ChrI
A0J47_RS04485	Glutaminase GlsA	–2.99	–1.58	4.10E-17	ChrI
A0J47_RS16645	Glycine cleavage system aminomethyltransferase GcvT	–4.54	–2.18	1.08E-156	ChrII
A0J47_RS10545	Acetolactate synthase I/II/III large subunit AlsS	–3.84	–1.94	5.49E-72	ChrI
A0J47_RS14750	Hippurate hydrolase HipO	–5.31	–2.41	6.95E-76	ChrII
***Compatible solute uptake systems***					
A0J47_RS18900	Glycine betaine/proline transport system substrate-binding protein	–10.69	–3.42	1.02E-165	ChrII
A0J47_RS18905	Glycine betaine/proline transport system permease protein	–5.00	–2.32	3.06E-31	ChrII
A0J47_RS18910	Glycine betaine/proline transport system ATP-binding protein	–3.36	–1.75	1.76E-51	ChrII
A0J47_RS08670	Betaine/Carnitine/Choline Transporter (BCCT) family transporter	–11.85	–3.57	5.68E-288	ChrI
A0J47_RS07020	Betaine/Carnitine/Choline Transporter (BCCT) family transporter	–4.80	–2.26	2.82E-198	ChrI
***Histamine production***					
A0J47_RS13250	Histidine-histamine antiporter HdcT	–3.98	–1.99	3.24E-28	ChrI
A0J47_RS13255	Histidine decarboxylase HdcA	–2.76	–1.46	1.66E-33	ChrI
A0J47_RS13260	Histidine-tRNA ligase HisRS	–1.59	–0.67	8.24E-13	ChrI
***Peptidases***					
A0J47_RS07600	M20 family metallopeptidase	–26.89	–4.75	0	ChrI
A0J47_RS13435	U32 family peptidase	–8.41	–3.07	1.55E-119	ChrI
A0J47_RS19220	C69 family dipeptidase	–5.32	–2.41	1.27E-78	ChrII
***Porins, permeases, and transporters***					
A0J47_RS05530	Outer Membrane Protein C	–12.49	–3.64	0	ChrI
A0J47_RS14790	Maltoporin LamB	–11.89	–3.57	9.44E-172	ChrII
A0J47_RS06290	Uncharacterized membrane protein YjiH	–12.44	–3.64	1.67E-24	ChrI
A0J47_RS08595	C4-dicarboxylate transporter DcuC	–9.03	–3.17	2.11E-111	ChrI
***Hypothetical and uncharacterized proteins***					
A0J47_RS17280	Hypothetical protein	–139.01	–7.12	0	ChrII
A0J47_RS17285	Hypothetical protein	–84.29	–6.40	0	ChrII
A0J47_RS12415	Helix-turn-helix domain-containing protein	–97.17	–6.60	1.31E-288	ChrI
A0J47_RS10160	Hypothetical protein	–24.70	–4.63	0	ChrI
A0J47_RS04560	Hypothetical protein	–24.69	–4.63	1.10E-94	ChrI

^*a*^ ChrI, chromosome I; ChrII, chromosome II.

**TABLE 2:** List of selected DEGs significantly upregulated at 1% NaCl

Locus_tag	Product/function	FC	Log_2_FC	*P*-value	Location^*a*^
***Virulence and antimicrobial resistance***					
A0J47_RS13280	PirA-like	348.13	8.44	0	ChrI
A0J47_RS13275	PirB-like	83.44	6.38	0	ChrI
A0J47_RS20355	Pore-forming toxin PhlyP	11.13	3.48	0	pPHDD1
A0J47_RS10995	Pore-forming toxin PhlyC	2.32	1.22	1.29E-59	ChrI
A0J47_RS20350	Damselysin	11.01	3.46	0	pPHDD1
A0J47_RS20585	Outer membrane protein OmpU	3.70	1.89	9.76E-100	pPHDD1
A0J47_RS20130	RNAase toxin Ntox44	4.77	2.25	1.14E-89	pPHDD1
A0J47_RS20325	AcrB/MacB-like ABC transporter ATP-binding protein	3.64	1.86	1.78E-153	pPHDD1
A0J47_RS20340	TolC family protein	3.60	1.85	6.53E-189	pPHDD1
A0J47_RS20345	AcrA/MacA-like membrane fusion protein	3.72	1.90	9.20E-173	pPHDD1
A0J47_RS20135	PAAR domain-containing protein	3.55	1.83	7.77E-79	pPHDD1
A0J47_RS11245	Putative lipoprotein	9.43	3.24	0	ChrI
A0J47_RS20555	TonB-dependent transferrin receptor Vep20-like	9.50	3.25	1.08E-220	pPHDD1
A0J47_RS20550	Serum resistance protein Vep07-like	4.69	2.23	2.21E-150	pPHDD1
A0J47_RS03660	Fe^3+^ ABC transporter substrate-binding protein FbpA	18.74	4.23	0	ChrI
A0J47_RS11430	Fe^2+^ transporter permease subunit FeoB	7.40	2.89	4.37E-197	ChrII
A0J47_RS11435	Fe^2+^ transport protein FeoA	5.98	2.58	6.13E-73	ChrII
A0J47_RS14665	TonB-dependent siderophore receptor FhuE	7.05	2.82	0	ChrII
***Protein export and secretion***					
A0J47_RS19585	Preprotein translocase subunit SecD	8.10	3.02	0	ChrII
A0J47_RS00865	Type II secretion system protein EpsJ	2.88	1.52	2.55E-50	ChrI
A0J47_RS00870	Type II secretion system protein EpsI	3.21	1.68	1.14E-34	ChrI
A0J47_RS00875	Type II secretion system protein EpsH	4.66	2.22	1.06E-91	ChrI
A0J47_RS00880	Type II secretion system protein EpsG	4.54	2.18	2.82E-198	ChrI
A0J47_RS00885	Type II secretion system protein EpsF	3.55	1.83	2.28E-88	ChrI
***Flagellar motility and chemotaxis***					
A0J47_RS12245	Chemotaxis protein CheV	1.69	0.75	3.38E-53	ChrII
A0J47_RS12040	Chemotaxis response regulator CheY	2.69	1.43	3.47E-80	ChrI
A0J47_RS05380	Methyl-accepting chemotaxis protein	2.58	1.37	8.54E-152	ChrI
A0J47_RS12030	Chemotaxis protein histidine kinase CheA	2.42	1.28	4.93E-56	ChrI
A0J47_RS12035	Chemotaxis regulator CheZ	2.42	1.28	2.59E-51	ChrI
A0J47_RS12075	Flagellar biosynthetic protein FliQ	2.31	1.21	3.72E-07	ChrI
A0J47_RS12200	Flagellar P-ring protein precursor FlgI	1.98	0.98	4.34E-26	ChrI
A0J47_RS12210	Flagellar basal-body rod protein FlgG	1.84	0.88	2.08E-20	ChrI
***Translation, ribosomal structure, and biogenesis***					
A0J47_RS00405	30S ribosomal protein S6-L-glutamate ligase RimK	52.24	5.71	0	ChrI
A0J47_RS16560	2OG-Fe(II) oxygenase	11.78	3.56	3.93E-161	ChrII
A0J47_RS00505	tRNA-dihydrouridine synthase B DusB	6.43	2.69	1.13E-196	
A0J47_RS06710	50S ribosomal protein L32 RpmF	4.73	2.24	1.70E-29	ChrI
A0J47_RS07485	Small subunit ribosomal protein S1 RpsA	4.29	2.10	3.66E-195	ChrI
A0J47_RS02550	50s ribosomal protein L31 RpmE	3.85	1.94	2.64E-102	ChrI
A0J47_RS09500	50S ribosomal protein L25 RplY	4.71	2.24	0	ChrI
***Metabolism and transport***					
A0J47_RS17020	Glutathione synthase GshB	38.42	5.26	0	ChrII
A0J47_RS15265	Class I SAM-dependent methyltransferase	29.87	4.90	1.43E-302	ChrII
A0J47_RS12810	Myo-inositol-1(or 4)-monophosphatase ShuB	26.88	4.75	0	ChrI
A0J47_RS08575	Agmatinase SpeB	11.13	3.48	0	ChrI
A0J47_RS17735	Gamma-glutamylputrescine oxidase	9.63	3.27	1.30E-244	ChrII
A0J47_RS01525	Glutamine-fructose-6-phosphate transaminase GlmS	7.50	2.91	9.23E-41	ChrI
A0J47_RS17685	Cysteine desulfurase	7.34	2.87	3.41E-147	ChrII
A0J47_RS17170	Carboxynorspermidine decarboxylase	4.47	2.16	1.99E-118	ChrII
A0J47_RS17175	Carboxynorspermidine synthase	5.42	2.44	0	ChrII
***Transporters***					
A0J47_RS09135	Spermidine/putrescine binding protein PotD2	9.02	3.17	2.40E-96	ChrI
A0J47_RS10120	Energy-coupling factor ATP-binding protein EcfA	8.28	3.05	4.99E-268	ChrI
A0J47_RS01035	7-cyano-7-deazaguanine/7-aminomethyl-7-deazaguanine transporter YhhQ	22.65	4.50	0	ChrI
A0J47_RS11695	TolC family protein CusC	9.73	3.28	8.73E-73	ChrI
A0J47_RS07645	GPR1/FUN34/yaaH putative acetate transporter	23.91	4.58	0	ChrI
A0J47_RS19570	NupC/NupG family nucleoside CNT transporter	9.46	3.24	7.88E-273	ChrII
A0J47_RS13535	Magnesium transporter MgtE	5.35	2.42	3.37E-182	ChrI
A0J47_RS06610	Na+/H+-dicarboxylate symporter	7.76	2.96	1.58E-55	ChrI
A0J47_RS10440	Na+-driven multidrug efflux pump (MATE)	6.13	2.62	1.92E-152	ChrI
A0J47_RS15280	MFS transporter	32.63	5.03	0	ChrII
A0J47_RS08650	MFS transporter	17.63	4.14	0	ChrI
A0J47_RS15440	MFS transporter	6.17	2.62	3.73E-150	ChrII
***Peptidases***					
A0J47_RS12990	Putative protease YegQ	11.93	3.58	0	ChrI
A0J47_RS08645	Peptidoglycan DD-metalloendopeptidase	9.97	3.32	0	ChrI
A0J47_RS18555	Trypsin-like serine protease	5.67	2.50	5.84E-262	ChrII
A0J47_RS03380	Do family serine endopeptidase	4.26	2.09	9.46E-17	ChrI
***Transcriptional regulators***					
A0J47_RS00510	DNA-binding transcriptional regulator Fis	5.81	2.54	5.61E-172	ChrI
A0J47_RS12800	Fe-S cluster assembly transcriptional regulator IscR	5.61	2.49	1.82E-256	ChrI
A0J47_RS01530	DeoR family transcriptional regulator	5.51	2.46	1.99E-21	ChrI
A0J47_RS08475	LysR family transcriptional regulator	2.78	1.47	2.60E-51	ChrI
***Histone acetylation and tRNA binding/modification***					
A0J47_RS19900	Histone acetyltransferase HPA2	17.18	4.10	1.21E-208	ChrII
A0J47_RS09065	tRNA 2-thiocytidine(32) synthetase TtcA	6.59	2.72	0	ChrI
A0J47_RS00505	tRNA dihydrouridine synthase DusB	6.43	2.69	1.13E-196	ChrI
A0J47_RS11195	tRNA 5-methoxyuridine(34)/uridine 5-oxyacetic acid(34)synthase CmoB	5.17	2.37	1.27E-218	ChrI
***Hypothetical and uncharacterized proteins***					
A0J47_RS17025	Flavohemoglobin expression-modulating QEGLA motif protein	27.51	4.78	0	ChrII
A0J47_RS17005	Hypothetical protein	18.64	4.22	0	ChrII
A0J47_RS10190	Hypothetical protein	13.27	3.73	1.35E-08	ChrI
A0J47_RS10185	Hypothetical protein	11.90	3.57	1.16E-55	ChrI
A0J47_RS19195	Uncharacterized membrane protein	9.61	3.27	3.45E-68	ChrII
A0J47_RS19225	DUF3316 domain-containing protein	9.59	3.26	5.71E-162	ChrII
A0J47_RS20565	Uncharacterized protein	4.78	2.26	9.38E-172	pPHDD1

^*a*^ ChrI, chromosome I; ChrII, chromosome II; pPHDD1, virulence plasmid.

### Salinity changes slightly impact cell morphology and capsule production in *Pdd* RM-71

Comparative analysis of the transcriptomes revealed that some genes involved in cell shape regulation were upregulated at low salt (Table S2), including the rod shape-determining protein RodA (A0J47_RS14950) and the peptidoglycan (PG) glycosyltransferase MrdB (A0J47_RS05095), both part of the shape, elongation, division, and sporulation (SEDS) family (48). In line with these findings, the *V. cholerae* gene (*pbpB*, VCA0870) homologous to *Pdd* RM-71 D-alanyl-D-alanine endopeptidase PbpG (A0J47_RS09265) was induced in the absence of NaCl and suggested to be involved in PG modulation under no salt (6, 49).

**Fig 4:**
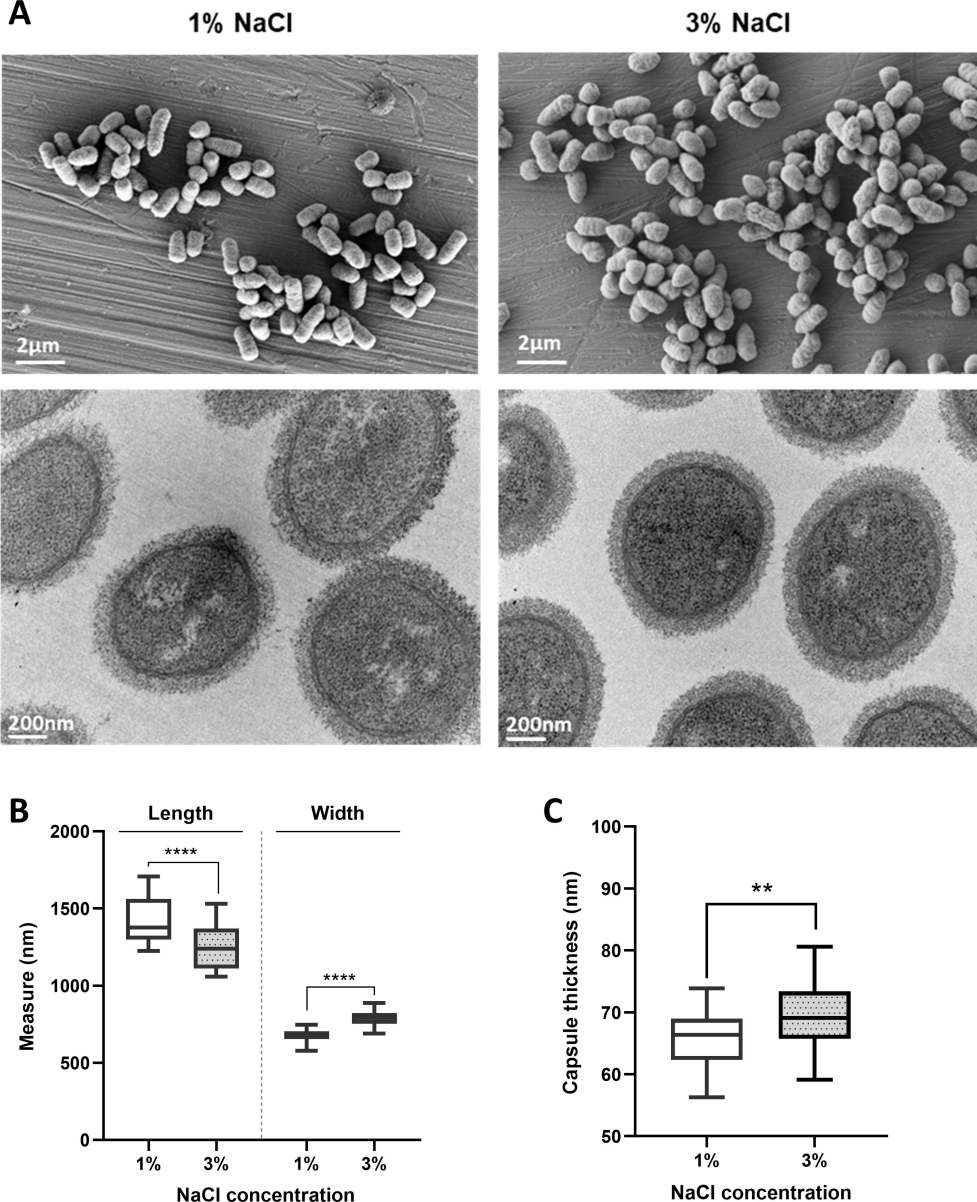
(**A**) Cell morphology and polysaccharide capsule of *Pdd* RM-71 grown at 1% NaCl and 3% NaCl, assessed by SEM (top panel) and TEM (bottom panel), respectively. (**B**) Box plot showing cell length and width of *Pdd* RM-71 cells at two assayed salinities. Cells were significantly longer and narrower at 1% NaCl. (**C**) Capsule thickness (in nanometer) of *Pdd* RM-71 at both salinities. Capsule was more compact and significantly thicker at 3% NaCl. Statistical difference was assessed by Student’s *t*-test: ****P < 0.0001, **P < 0.01.

Cell morphology of RM-71 was assessed by SEM and TEM. SEM images revealed that cells grown at low salt exhibited a significantly more elongated shape and were narrower than cells at 3% NaCl (Fig. 4A and B). Polysaccharide capsule constitutes a virulence factor in *Pdd* RM-71, playing a role in protection against fish serum, and a gene cluster participating in capsule biogenesis has been functionally characterized (28). In the present study, we found that capsule biogenesis genes are slightly upregulated at 1% NaCl (Fig. 3, chromosome I panel; Table S2), with FC values ranging from 1.7 to 3.57. However, TEM analysis revealed a significant, albeit moderate increase in capsule thickness at 3% NaCl (average thickness of 65.55 and 69.43 nm for 1% and 3% NaCl, respectively) (Fig. 4C). These apparently contradictory observations suggest the participation of additional, yet-unknown regulatory mechanisms in *Pdd* capsule production. The presence of fully capsulated cells at either salinity condition indicates that *Pdd* expresses the polysaccharide capsule during its environmental lifestyle as well as during an infection. While the protective role of the capsule during animal infection has been previously established in *Pdd* (28), the function of the capsule during the free-living lifestyle is unknown. Previous studies have shown that *Pdd* can be predated by *Bdellovibrio bacteriovorus* (50) and by heterotrophic marine flagellates and microzooplankton (51, 52). It can be speculated that the capsule may play a protective role against predation in the marine environment. It is pertinent to note that not all *Pdd* strains are capsulated (A. V. Barca, A. do Vale, and C. R. Osorio, unpublished data), emphasizing the need for in-depth studies to clarify the role of capsule in predation scenarios.

### Energy production and carbohydrate metabolism are upregulated at 3% NaCl

*Pdd* is a highly heterogeneous subspecies, and the populations causing disease in marine animals are considered to be of multiclonal nature (25, 53). Nevertheless, there are conserved biochemical features that define the subspecies. *Pdd* is heterotrophic, non-nitrogen fixing, facultative anaerobic capable of reducing nitrate to nitrite, ferments glucose with the production of gas, produces arginine deiminase, and most strains produce urease (47). The comparative study of the *Pdd* transcriptomic profiles revealed the upregulation, at 3% NaCl, of genes involved in energy production and carbohydrate and amino acid metabolism (Fig. 2; Table 1), being notable that the genes encoding F_0_F_1_ ATP synthase subunits were among the top-20 upregulated genes. This upregulation of the ATP synthesis machinery might account, at least in part, for the increased growth observed at 3% NaCl (Fig. 1A). Enzymes involved in aerobic and anaerobic respiration were markedly upregulated at 3% NaCl including the *frdABCD* fumarate reductase operon, the *asrABC* sulfite reductase operon, and nitrite reductase subunits (Table 1). The enhanced expression of aerobic and anaerobic respiration enzymes under high salt has also been reported in *Shewanella* sp. and *Escherichia coli* (54, 55).

We observed a strong upregulation of genes involved in response to reactive nitrogen species. Noteworthy, the gene encoding a hydroxylamine reductase is upregulated 58-fold at 3% NaCl. This enzyme, homologous to hybrid cluster protein Hcp of *E. coli*, catalyzes the reduction of hydroxylamine to NH_3_ and H_2_O and is predicted to have a role in protection against nitrosative stress (56). In addition, it was observed the upregulation of a nitrous oxide stimulated promoter family protein homologous to *E. coli* YgbA. These two genes are under the control of the regulatory protein NsrR, a nitric oxide–sensitive regulator of transcription, characterized in *E. coli* (57) and whose homolog in *Pdd* identified in the present study (A0J47_RS00170) is also upregulated at 3% NaCl (Table 1). These findings clearly suggest that seawater salinity boosts nitrogen metabolism in *Pdd*, leading to the potential activation of nitrosative stress response mechanisms.

The cytochrome c-type subunit TorY (56% amino acid identity to *E. coli* TorY/Yeck) and a molybdoenzyme reductase (68% amino acid identity to *E. coli* TorZ/BisZ) are 62- and 16-fold upregulated at seawater salinity, respectively (Table 1). This system was first described in *E. coli* as an additional anaerobic respiration system that uses trimethylamine *N*-oxide (TMAO) and biotin sulfoxide as alternative electron acceptors (58). Of note, it has been reported in a previous study that *Pdd* is capable of reducing TMAO to TMA (trimethylamine) (59). Additionally, genes involved in glucolysis/gluconeogenesis, pyruvate and acetate metabolism were upregulated at 3% NaCl (Table 1). So are genes encoding an acetate kinase and an alcohol dehydrogenase, suggesting that acetate production is enhanced at seawater salinity in *Pdd*. The upregulation of genes involved in acetate production has been reported in *V. vulnificus* as an antipredation strategy against protozoans (60).

Considering all the functions upregulated at 3% NaCl, energy production systems including aerobic and anaerobic respiration systems as well as carbon metabolism might be of high priority when *Pdd* grows in a free-living state. The enhanced expression of these gene categories was also observed in *P. damselae* subsp. *piscicida* when shifting from low to high salt (61) and in *E. coli* in response to growth in seawater (55).

### Growth at 3% NaCl upregulates genes involved in the uptake of compatible solutes and in the use of trehalose and fructose as carbon sources

To thrive in high-osmolarity environments, bacterial cells accumulate compatible solutes (osmolytes) in the cytoplasm, via either uptake or biosynthesis (62, 63). Osmolytes can be sugars (as trehalose), amino acids (proline and glutamine), and quaternary amines (glycine betaine, choline). Acquisition of compatible solutes from the environment is preferable in terms of cell economy, since the biosynthesis of osmolytes is energetically costly. In line with this, we observed that the growth of *Pdd* at 3% NaCl enhanced the expression of uptake systems for compatible solutes (Fig. 2; Table 1), namely the chromosome II-encoded ProU system for the uptake of glycine betaine and L-proline. The enhanced expression of *proXVW* genes under hyper-osmotic conditions has been reported in *V. parahaemolyticus* (8) and *V. vulnificus* (64).

**Fig 5:**
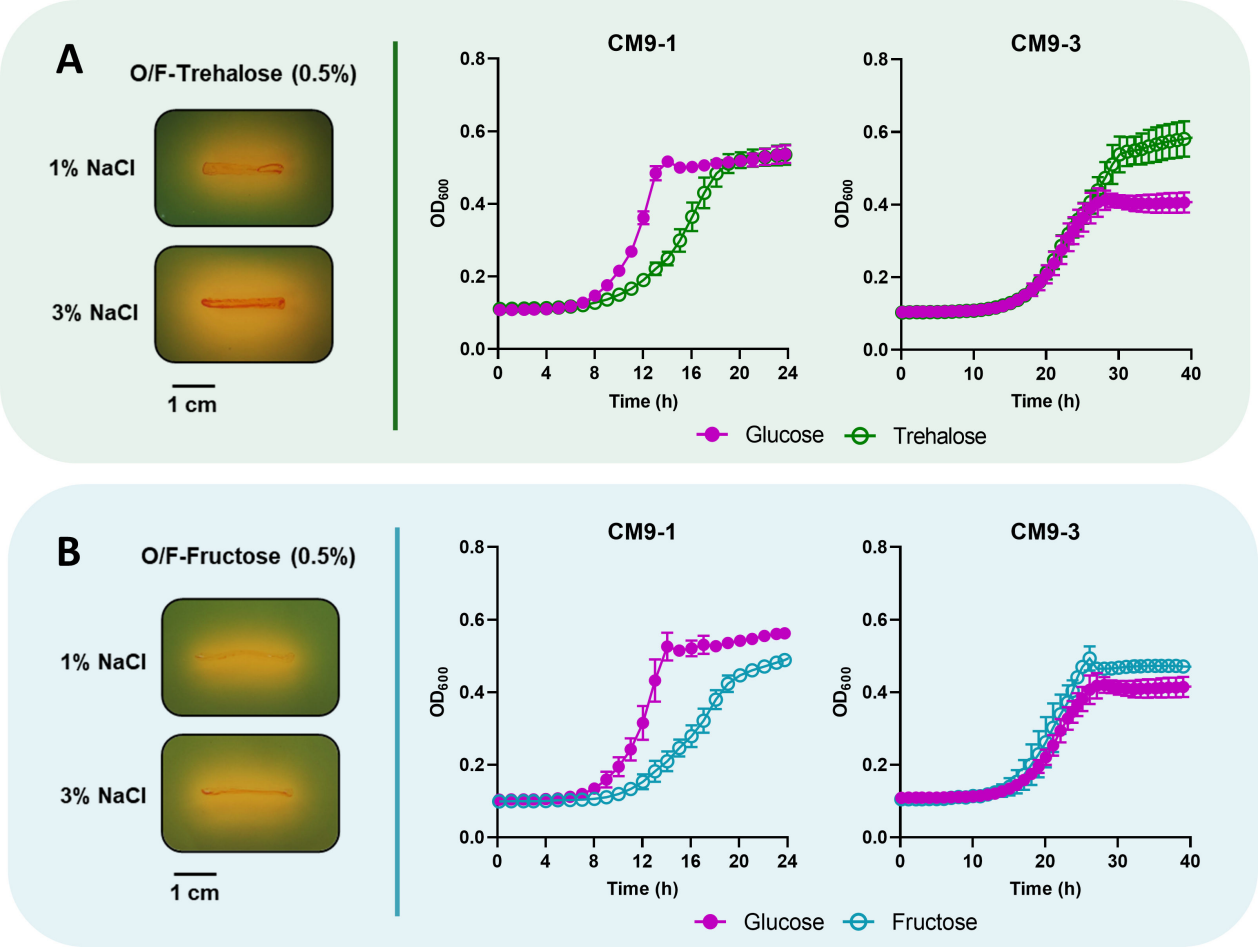
Acid production of *Pdd* RM-71 (left panels) by fermentation of trehalose (**A**) and fructose (**B**) under 1% NaCl and 3% NaCl in oxidation–fermentation (O/F) medium. Growth curves of RM-71 in CM9 medium (right panels) supplemented with 0.5% glucose compared with the growth in CM9 medium with 0.5% trehalose (A) or 0.5% fructose (B) at 1% NaCl and 3% NaCl. Data are presented as mean ± SD from three biological replicates and two independent experiments.

Albeit the type strain of *Pdd* (ATCC33539) was reported to be unable to use trehalose (47), we here found three genes of the trehalose-specific PTS (phosphotransferase system) (*treB*, *treC*, and *treR*) in the genome of RM-71 (genes absent in the type strain) that were 2- to 15-fold upregulated at high salt. This observation suggests that *Pdd* RM-71 may use trehalose as a compatible solute (osmolyte) similar to what has been described in other members of the family *Vibrionaceae* (63), and trehalose may also be used as a carbon source (65). When we streaked *Pdd* RM-71 on plates of O/F (oxidation–fermentation) medium supplemented with trehalose, we observed a visibly higher production of acid at 3% NaCl than at 1% (Fig. 5A). To quantitatively measure the possible preference of trehalose versus glucose at 3% NaCl by *Pdd*, we monitored the growth of RM-71 at each salinity condition in the presence of either glucose or trehalose, using CM9 (minimal medium M9 supplemented with casamino acids). Interestingly, at 3% NaCl trehalose conferred higher growth levels than glucose, whereas at 1% NaCl glucose was the carbon source that allowed for higher growth rates (Fig. 5A).

An early study reported that the type strain of *Pdd* encodes a putative PTS system for fructose utilization (66). Our RNA-seq data also revealed that 3% NaCl causes the upregulation of genes of a PTS system for fructose (Table 1). Similar to trehalose, we observed that the growth of *Pdd* RM-71 at 3% NaCl in presence of fructose is higher than in presence of glucose, whereas at 1% NaCl glucose is the preferred carbon source and allows higher growth rates (Fig. 5B). All these observations clearly indicate that at seawater salinity, the use of trehalose and fructose is enhanced in comparison to glucose. This poses a notable biological significance, considering that trehalose and fructose are highly abundant sugars in marine environments (67 - 69). Trehalose constitutes the main sugar in some species of macroalgae (70) and is also a major oligosaccharide in the hemolymph of some crustaceans (71). Fructose availability would come mainly from the hydrolysis of sucrose, which is abundant in seagrass meadows (69, 72) and is produced by green algae and cyanobacteria (73).

### Growth at 3% NaCl enhances tolerance to kanamycin, vancomycin, and ampicillin

**Fig 6:**
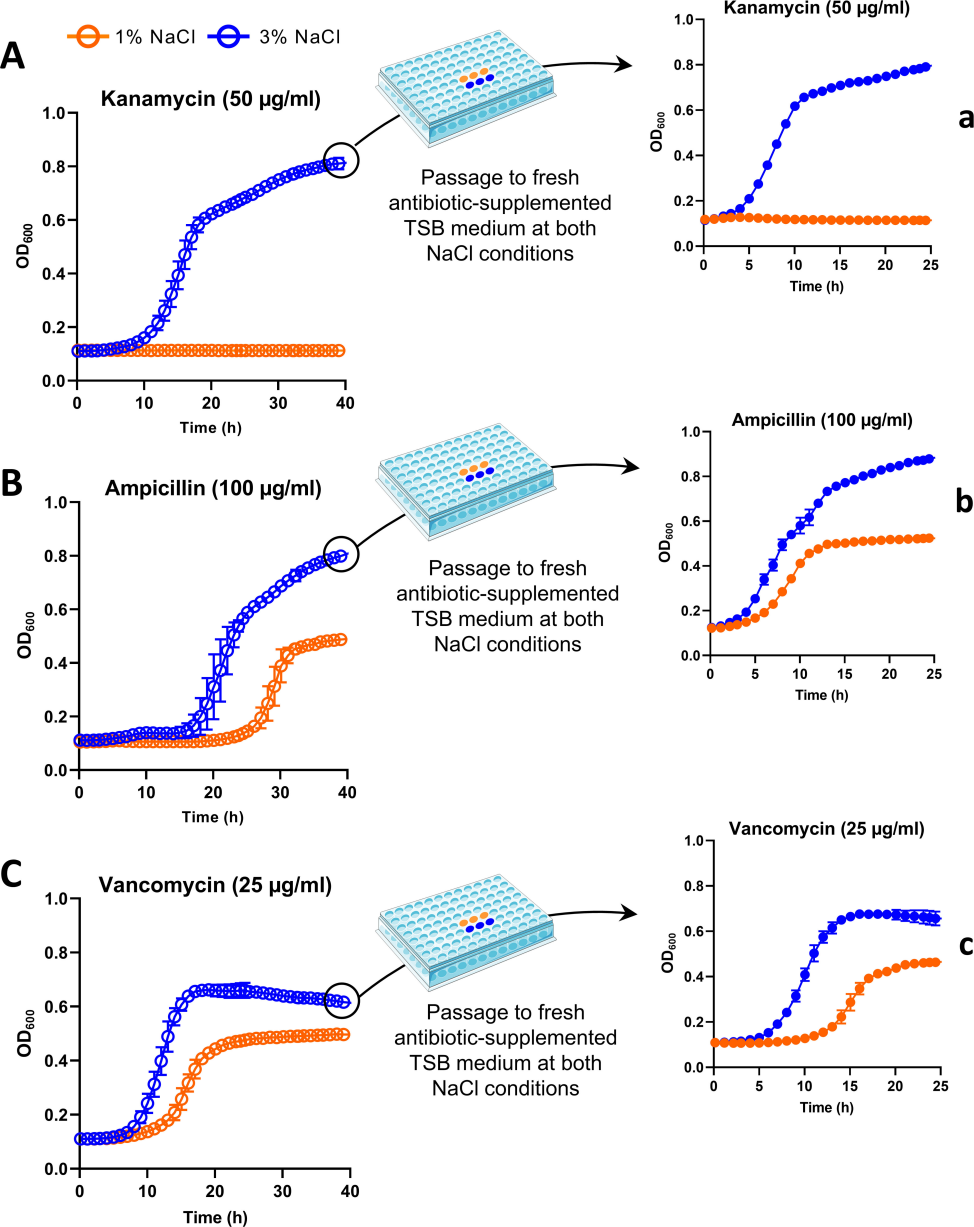
NaCl changes impact antibiotic resistance in *Pdd.* Growth curves of RM-71 in TSB-1% NaCl and 3% NaCl supplemented with kanamycin (50 µg/mL) (**A**), ampicillin (100 µg/mL) (**B**), and vancomycin (25 µg/mL). After 40 hours, RM-71 cells grown at 3% NaCl in each antibiotic were passaged to fresh antibiotic-supplemented TSB medium at both NaCl conditions (a, b, and c). Data are presented as mean ± SD (*n* = 2).

We found that *Pdd* RM-71 grown at standard conditions of 1% NaCl exhibits intrinsic resistance to ampicillin and vancomycin, similar to other species of the family *Vibrionaceae* (74, 75) while it is sensitive to kanamycin (Fig. 6). Surprisingly, *Pdd* RM-71 showed increased tolerance to kanamycin, ampicillin, and vancomycin at 3% NaCl compared to 1% NaCl (Fig. 6). The most drastic increase was observed with kanamycin, as RM-71 at 3% NaCl grows up to an OD_600_: 0.8 with antibiotic concentrations (50 µg/mL) that resulted inhibitory at 1% NaCl. To demonstrate that the NaCl-promoted antibiotic tolerance constitutes a salinity-dependent response of *Pdd* cells and is not a consequence of the selection of stable mutations conferring antibiotic resistance, late stationary-phase cells grown at 3% NaCl in the presence of each antimicrobial were passaged to fresh antibiotic-supplemented media at the two NaCl concentrations, and the growth was monitored. As shown in Fig. 6, the phenotype of increased tolerance at 3% NaCl with respect to 1% NaCl is maintained for the three antibiotics tested, suggesting that the NaCl-induced antibiotic tolerance is due to a regulatory, adaptive mechanism in *Pdd*.

The induced tolerance to antimicrobials under high salt has been reported in *Vibrios* (76, 77), but the underlying molecular mechanisms are poorly understood. Several mechanisms have been described for beta-lactam resistance in *Vibrios* (78, 79). Contradictorily, most genes categorized in beta-lactam resistance (ko01501 pathway in KEGG), such as penicillin-binding proteins (PBPs), multidrug efflux pumps (A0J47_RS15280, A0J47_RS08650), and outer membrane (OM) proteins were upregulated at low salt (Table 2; Table S2). However, it is pertinent to clarify that the ligands of these putative multidrug pumps remain completely undeciphered in *Pdd*, and they may serve functions different from antibiotic resistance.

We identified genes whose upregulation at 3% NaCl could account for the increased resistance to ampicillin. These include a gene for a D-alanyl-D-alanine carboxypeptidase (A0J47_RS09080) (Pbp5) (FC: −1.94) (Table S2) whose deletion in *E. coli* led to an increase in ampicillin susceptibility (80). In addition, previous research reported that some PG peptidases from *V. cholerae*, particularly ShyA, are critical to maintain cell viability on treatment with beta-lactams (79). Supporting our data on growth in the presence of ampicillin at 3% salt, a PG endopeptidase (A0J47_RS18260) homolog to *V. cholerae* ShyA (52% ID, VCA0079) was induced under high salt in *Pdd* (FC: −3.01).

Mechanisms of aminoglycoside resistance include the acquisition of resistance plasmids, changes in membrane permeability, and/or active efflux of antibiotics (81). We observed that the expression of a Na^+^-driven multidrug efflux pump (A0J47_RS00420) is upregulated at 3% NaCl in *Pdd* (FC: −1.82). This protein is homologous to *V. cholerae* VcmA (VC1540; 27% identity, 65% coverage), a protein responsible for increased resistance to kanamycin (82).

Regulation of bacterial metabolism has been identified as an additional mechanism that supports antibiotic resistance in *Vibrio* and other species (83 - 85). Indeed, a recent work has demonstrated that NaCl negatively regulates proton motive force (PMF) in *Vibrio alginolyticus*, leading to a reduction in aminoglycoside uptake and a subsequent increase in antibiotic resistance at high salt (86). Nevertheless, our transcriptomic data did not reveal a clear contribution of NaCl to PMF changes and its possible impact on aminoglycoside uptake.

Gram-negative OM is an extraordinary barrier to high-molecular-weight antibiotics, such as vancomycin (87). A previous research identified the inner membrane protein VigA conferring vancomycin resistance in *V. cholerae* (74), but no homologs of VigA were identified in the genome of *Pdd* RM-71. Vancomycin exerts its action by binding to D-Ala-D-Ala residues from the bacterial PG, affecting normal cell growth. The enzyme D-ala-D-ala ligase (Ddl) catalyzes the ligation of these residues in the assembly of the bacterial PG, but alternative Ddl ligases (Van ligases) produce other PG precursors with lower affinity for the antibiotic (88). Interestingly, we found a gene (A0J47_RS04780) upregulated (FC: −1.73) at high salt in *Pdd* (Table S2), whose protein shares 33% identity (BlastP searches in CARD: The Comprehensive Antibiotic Resistance Database) with VanL of *Enterococcus faecalis* (ABX54687.1), a ligase that synthesizes the alternative D-Ala-D-Ser substrate with less affinity for vancomycin (89). The contribution of this gene in the enhanced vancomycin resistance observed at high salt has not been previously reported in *Pdd* and would surely deserve future investigations.

*Pdd* is sensitive to antimicrobial peptides produced by marine fish and arthropoda, such as hepcidin (90), pleurocidin-amide and tachyplesin (91), and piscidin (41). We here tested the possible effect of salinity on the sensitivity of *Pdd* RM-71 to piscidin but did not find differences at 3% NaCl versus 1% NaCl (Fig. S3). Considering that piscidin is believed to act at the level of the cell membrane (92), our results suggest that salinity does not cause appreciable differential regulation of cell functions that might be involved in susceptibility to this fish-derived, antimicrobial peptide.

### Salt-mediated regulation of amino acid metabolism highlights major changes in arginine catabolism

**Fig 7:**
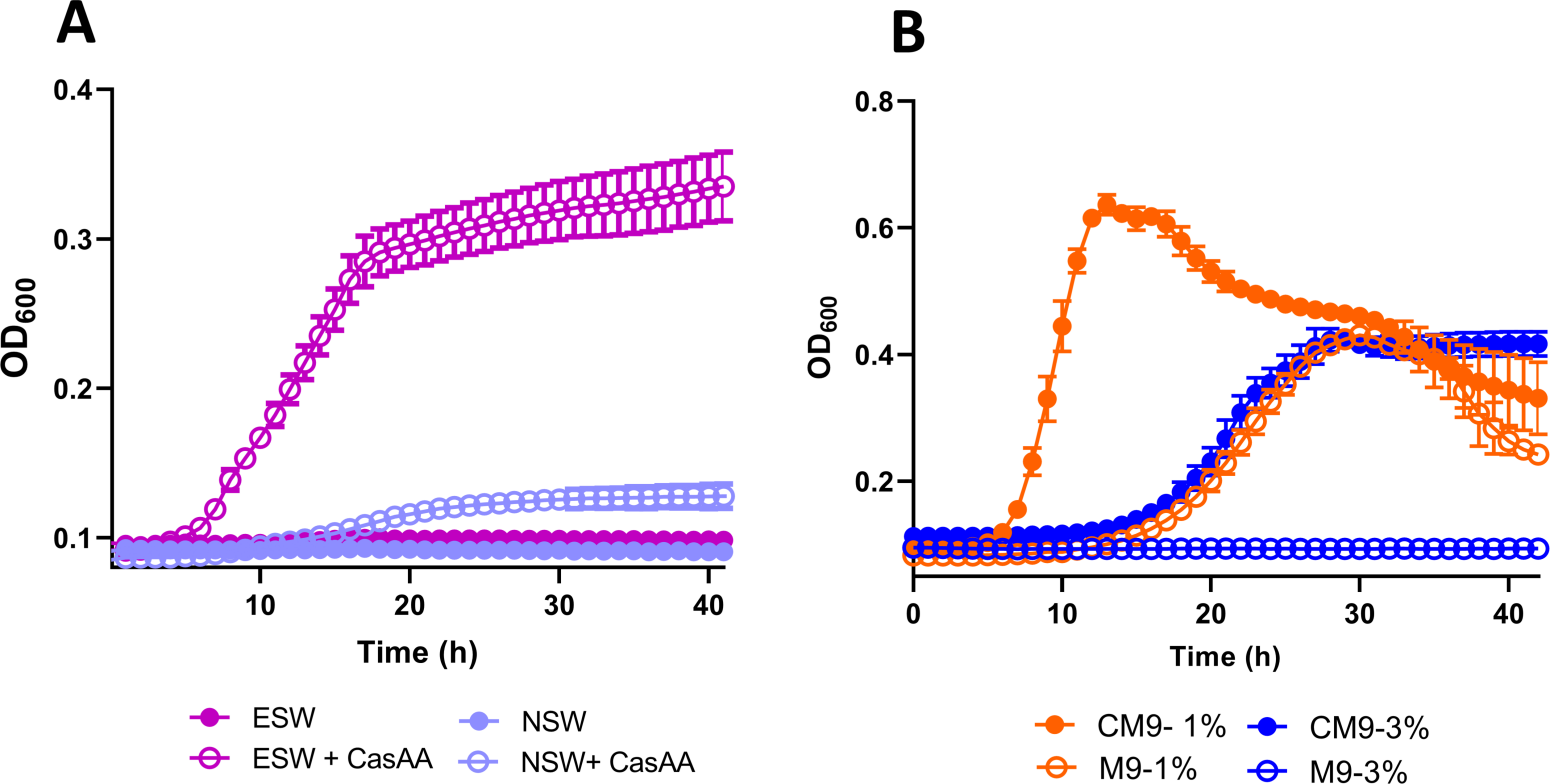
(**A**) Growth curves of *Pdd* RM-71 in plain coastal seawater (NSW) and eutrophicated seawater (ESW) with or without casamino acids (CasAA). (**B**) Growth of *Pdd* RM-71 in minimal medium M9 with 1% NaCl or 3% NaCl supplemented (CM9) or not (M9) with CasAA. The OD_600_ was monitored for 24 hours. Data are presented as mean ± SD from three biological replicates and two independent experiments.

One of the best-studied traits of the amino acid metabolism in *Pdd* is its high histamine-producing ability (>1,000 ppm), being able to release toxic levels of histamine in fish even under refrigeration temperatures (4°C) and hence posing a considerable risk for human health (93). Notably, the transcriptomics analysis revealed that at 3% NaCl *Pdd* upregulates the genes *hdcT*, *hdcA,* and *hisRS* involved in histamine production (Table 1), a finding consistent with histamine production taking place in decomposing fish in the marine environment and, hence, at salinities close to 3% NaCl. To better understand which amino acid pathways undergo significant changes in response to NaCl, salt-regulated pathways were identified by mapping DEGs to KEGG database. We observed a general upregulation of genes involved in amino acid pathways at 3% NaCl (Fig. S4) suggesting that amino acid metabolism is key to support bacterial growth and survival in a free-living lifestyle. The modulation of amino acid metabolism in response to NaCl has also been reported in *V. parahaemolyticus* (9) and *E. coli* (94). Though *Pdd* survives in seawater microcosms at 14°C and 22°C for longer than 1 year (95), we observed that natural coastal seawater does not support detectable growth of *Pdd* RM-71 (measured as an increase in optical density) after 40 hours, even when using ESW (Fig. 7A). Interestingly, the addition of casamino acids substantially improved growth but only in eutrophicated water (Fig. 7A). We also observed that *Pdd* RM-71 is capable of growing in minimal medium with glucose in presence of NH_4_^+^ ions as sole nitrogen source at 1% NaCl but not at 3% NaCl (Fig. 7B). This result suggests that inorganic nitrogen is not routinely used by *Pdd* under environmental conditions in seawater, but it is used under low-salt stress. All these observations suggest that *Pdd* thrives in organic matter–rich microenvironments but not so much in pelagic, nutrient-scarce seawater, and amino acid availability may constitute a limiting factor for the build up of *Pdd* populations in the environment. In a study of coastal vibrioplankton, it was found that the presence of *Pdd* was minor in comparison with, for instance, the *Vibrio splendidus* group (96). In fact, albeit *Pdd* is occasionally isolated from coastal seawater, it is more prevalent in nutrient-rich ecological niches and surfaces, such as gastrointestinal tracts of sharks and teleost fish (97, 98), fish gill microbiota (99), decomposing fish (100), and, especially, in the internal milieu of animal hosts suffering a *Pdd* infection, where it can constitute the major pathogen isolated, in many instances as pure culture (101).

**Fig 8:**
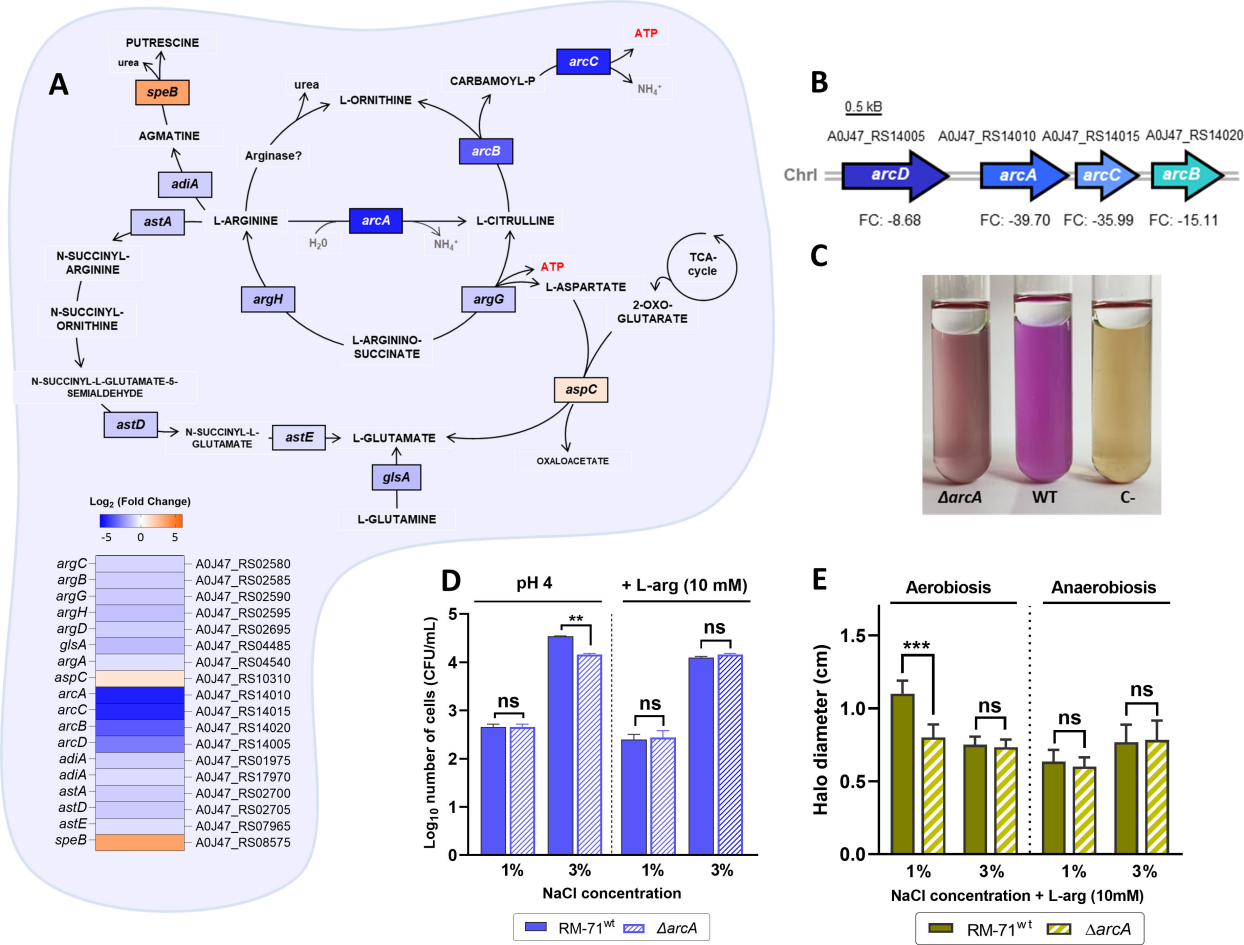
(**A**) Metabolic map showing differentially expressed genes (DEGs) related to arginine catabolism. DEGs are placed in the corresponding metabolic reaction with a color-based scale. Expression values are depicted in the heat map as Log_2_FC. Genes most upregulated at 3% NaCl (blue) correspond to the arginine deiminase pathway (*arcABCD*). Genes involved in the arginine succinyltransferase system (AST) were also upregulated at high salinity. (**B**) Gene map of the arginine deiminase system in RM-71 genome. The NCBI locus tag and the corresponding fold change (FC) value are displayed for each gene. (**C**) Moeller’s test results for arginine decarboxylation/deamination of ∆*arcA*, RM-71^wt^ (wild type), and non-inoculated control (C-). The light violet color observed in ∆*arcA* strain suggests the activity of other arginine-based alkalinizing enzymes. (**D**) Graph showing RM-71^wt^ and ∆*arcA* viability after 16 hours of incubation at pH 4 in TSB-1 and TSB-3 and supplemental L-arginine (10 mM). A Student’s *t*-test was used to assess statistical significance (***P < 0.001, **P < 0.01; ns, not significant). (**E**) Graph showing swimming motility diameters of RM-71 and ∆*arcA* in TSB-1 and TSB-3 supplemented with 10-mM L-arginine (L-arg) under aerobic and anaerobic conditions. ∆*arcA* motility is significantly reduced in the presence of L-arg at 1% NaCl. Data represent the mean ± SD from three biological replicates and two independent experiments.

*Pdd* is one of the few vibrios that produces arginine deiminase (also known as arginine dihydrolase), a key phenotypical test employed in taxonomy and identification of vibrios (102). In fact, *Pdd* (which was formerly known as *V. damsela*) was classically included in the “group *F* vibrios” or “group EF6” together with *Vibrio furnissii* and *Vibrio fluvialis* (103), positive for arginine deiminase. Through the arginine deiminase (ADI) pathway, L-arginine is converted to L-ornithine, yielding ATP, carbon dioxide, and ammonia through three metabolic steps (Fig. 8A). Intriguingly, our RNA-seq analysis revealed that the *arcACBD* operon encoding the ADS, responsible for L-arginine internalization and catabolism, is listed among the top upregulated genes at 3% NaCl, being *arcA* and *arcC* genes ca. 40-fold upregulated (Table 1; Fig. 8B). Additionally, genes of another L-arginine catabolic pathway, the arginine succinyltransferase system, were also upregulated at high salinity (Table 1).

The fitness advantages to *Pdd* in upregulating ADI pathway under marine-like salinity or, to put it the other way, to downregulate it under low salinity, remain unknown. To gain an insight into this, we deleted *arcA* gene in *Pdd* RM-71 by allelic exchange. Deletion of *arcA* caused a visible phenotypical change in the classical test for arginine deiminase, which detects alkalinization of the medium indicated by a purple coloration (Fig. 8C). This medium detects alkalinization caused by more than one enzymatic activity, including both arginine deiminase (also known as arginine dihydrolase) and arginine decarboxylase, and this may explain why the *arcA* mutant still produces residual purple color in this test. The *arcA* mutant did not show impairment for growth either in TSB medium at 1% NaCl and 3% NaCl (Fig. S5A) or in the presence of supplemented L-arginine (Fig. S5B) with respect to the parental strain.

The ADI pathway has a role in bacterial acid stress survival in many species of bacteria (104 - 106). We, therefore, aimed at investigating whether the mutation of *arcA* exerted any effect on resistance to acidic pH in *Pdd*. We first analyzed the growth of RM-71 in TSB-1 and TSB-3 at various pH levels (pH 7, pH 6, pH 5, and pH 4) and observed that pH 4 did not allow *Pdd* replication either at 1% NaCl or at 3% NaCl (data not shown). We next evaluated the viability of RM-71 and ∆*arcA* on acid stress (pH 4) in TSB-1 and TSB-3 supplemented or not with L-arginine (10 mM). Interestingly, the survival of RM-71 and ∆*arcA* strains after 16-hour exposure to acid conditions was ca. 3 orders of magnitude higher at 3% NaCl than at 1% NaCl (Fig. 8D), thus demonstrating that salinity has a major impact on *Pdd* survival at pH 4. In line with these findings, previous studies have demonstrated that high salinity increased *V. parahaemolyticus* survival against acid stress (107, 108). In addition, it was observed that the ∆*arcA* strain exhibited a slight, but significant, impairment for acid resistance in comparison to RM-71, suggesting a role of the ADI pathway on *Pdd* resistance to acidic conditions (Fig. 8D). This impairment was detected in TSB-3 with no additional L-arginine supplement but was not detected in presence of a surplus of L-arginine. This last observation suggests that an excess of arginine might allow other arginine-degrading pathways (e.g., the arginine decarboxylase system) to contribute to acid resistance and thus compensate for the absence of a functional ADI pathway.

*Pseudomonas aeruginosa* uses ADI activity to fermentatively generate ATP (109) and to maintain motility under anaerobiosis (110). We noted that the *arcA* mutant was not affected in swimming motility in anaerobiosis at either salinity condition but exhibited significant impairment under aerobiosis in the presence of supplemented L-arginine at 1% NaCl (Fig. 8E), a seemingly contradictory observation (ADI pathway genes were here found to be upregulated at 3% NaCl) that indicates that the involvement of the ADI pathway in *Pdd* motility needs additional investigation. Nevertheless, the observation that arginine deiminase is also upregulated in *Pdd* under iron-excess conditions (32) supports the hypothesis of a preferential role of this enzymatic activity during the free-living lifestyle versus the pathogenic lifestyle. It has been shown that uptake and respiration of L-arginine are maintained under nutrient starvation in *Vibrio* sp. (111). This strategy might be important for *Pdd* in its survival for long periods in low-nutrient seawater (95).

### Low salt triggers a virulence profile with a major impact on the upregulation of the T2SS-dependent secretome

Entry into the internal milieu of a vertebrate host leads to an abrupt drop in salinity. We found that 1% NaCl upregulates many gene functions potentially related to virulence. These functions include motility and chemotaxis, synthesis and uptake of polyamines, stress response mechanisms and efflux systems, and, notably, iron acquisition mechanisms and cytotoxins (Fig. 2; Table 2). The glutathione synthetase GshB was within the top upregulated functions at low salt, with an FC value of 38.4. Mutation of this gene has been associated with a strong impairment in colonization ability in *V. cholerae* (112). Previous studies reported the presence of polyamines putrescine, cadaverine, norspermidine, and spermidine in *Pdd* cells (93, 113, 114). We found that low salinity upregulates genes for the production of putrescine and norspermidine synthesis and transport (Table 2). The implication of spermidine and putrescine in the virulence of bacterial pathogens has been reported in several studies (115 - 117). However, the role of polyamines in *Pdd* pathobiology remains unknown.

The hallmarks of *Pdd* pathobiology in infected animals include the extensive hemorrhages and tissue damage caused by the secretion, in very high amounts, of the T2SS-dependent phospholipase D Dly and the two pore-forming toxins PhlyP and PhlyC (24). A pioneering study on *Pdd* toxin production reported that cytolytic activity was maximal when the bacteria were grown at 1% NaCl (118). In agreement with these findings, the plasmid-borne genes encoding Dly (*dly*, A0J47_RS20350) and PhlyP (*hlyA_pl_*, A0J47_RS20355) were 11-fold upregulated at low salt, whereas the *hlyA_ch_* gene encoding the chromosomal hemolysin PhlyC (A0J47_RS10995) was 2-fold upregulated (Table 2).

Unexpectedly, the list of top-upregulated genes at 1% NaCl was dominated by two genes encoding a small, 11-kDa protein (A0J47_RS13280) (PirA) and a putative δ-endotoxin (A0J47_RS13275) (PirB), respectively (Table 2). These two proteins are candidates to constitute a PirAB-like binary toxin for its similarity to the PirAB toxin produced by the strains of *V. parahaemolyticus* causing acute hepatopancreatic necrosis disease in crustaceans (119). However, the role of this putative PirAB-like toxin in *Pdd* remains unknown. The 11-kDa and δ-endotoxin proteins were previously identified in a study that characterized the T2SS-dependent secretome of *Pdd* RM-71 (27) and were subsequently confirmed as genes of the RstAB regulon, a two-component system that is a master positive regulator of virulence in *Pdd* (28). The genes for 11-kDa (PirA) and δ-endotoxin (PirB) are not ubiquitous in the subspecies. They occur in some *Pdd* isolates and are located within a highly variable region of the *Pdd* chromosome II, suggesting their acquisition by horizontal gene transfer (53).

This strong upregulation of the 11-kDa and δ-endotoxin genes under salinity conditions that mimic the hosts’ milieu prompted us to construct a knockout mutant of the larger protein (δ-endotoxin or PirB) and assess its impact in virulence for turbot, the host of *Pdd* RM-71. We found that deletion of the δ-endotoxin gene did not cause a detectable impairment in virulence (Fig. S6A). The δ-endotoxin mutant produced hemolytic activity at levels identical to the parental strain (Fig. S6B) and exhibited normal growth (Fig. S6C). As a comparison, a quadruple mutant of *Pdd* RM-71 defective in Dly, PhlyP, PhlyC, and PlpV cytotoxins, but with intact δ-endotoxin gene, was non-virulent under the conditions tested (Fig. S6A). These results suggest that the δ-endotoxin gene is expendable for maximal virulence of *Pdd* RM-71 in a turbot fish model. Notwithstanding, considering the broad host range of *Pdd*, it would be interesting to conduct future studies to assess the role of this putative PirAB-like toxin for other animal hosts, including fish and crustaceans.

**Fig 9:**
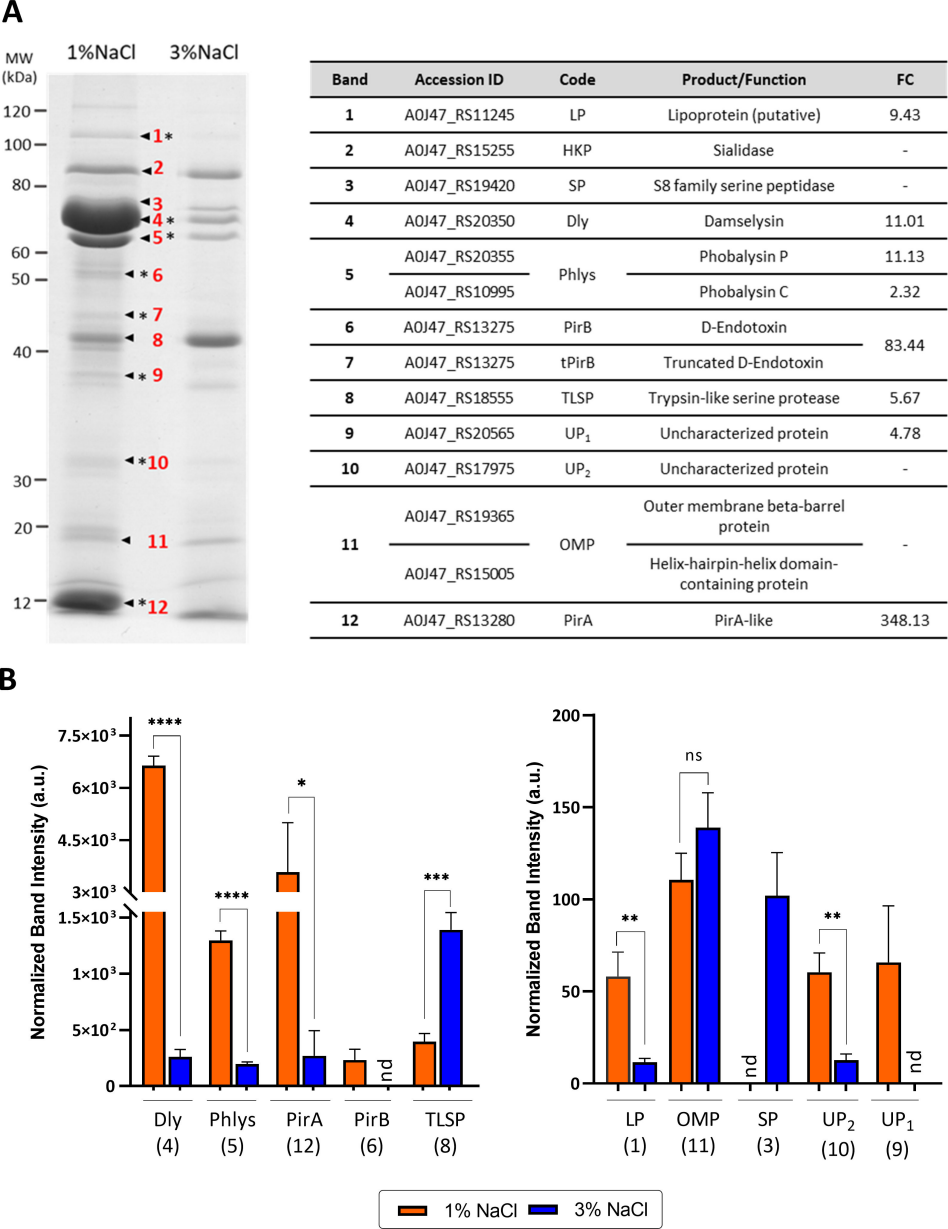
Salinity modulates the abundance of secreted proteins in *Pdd*. (**A**) Comparative analysis of SDS-PAGE profiles of culture supernatants from RM-71^wt^ at 1% NaCl and 3% NaCl. Previously identified protein bands (27) are numbered from 1 to 12. Bands with higher abundance at low salt are marked with an asterisk (*). Identification and tagging of the proteins is shown in the adjacent table. (**B**) Protein quantification results were obtained by densitometric analysis using ImageLab Software (Bio-Rad). Sialidase band (2, HKP) was used as a normalization control. Data are presented as mean ± SD of normalized protein intensity (expressed as a.u, arbitrary units) from three replicates over three independent experiments. The corresponding band on the SDS-PAGE gel shown in (**A**) is given in parentheses. nd, not detected. Statistical difference was assessed by Student’s *t*-test: ****P < 0.0001, ***P < 0.001, **P < 0.01, *P < 0.05.

**Fig 10:**
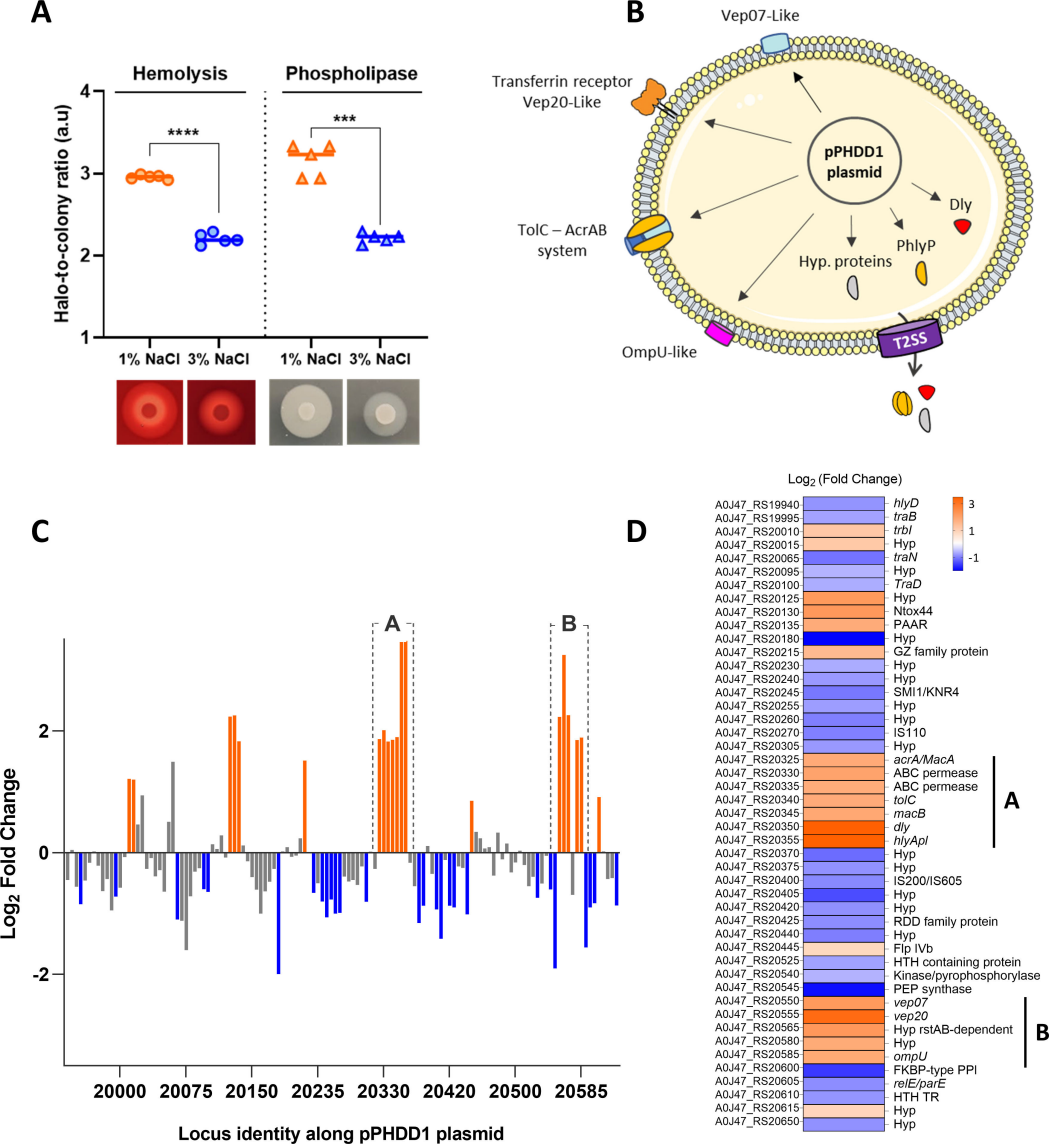
Differential gene expression patterns in the virulence plasmid pPHDD1 in response to changes in NaCl concentration. (**A**) Box plot showing hemolytic and phospholipase activities (expressed as a.u, arbitrary units, of the halo-to-colony ratio) of RM-71 at two NaCl conditions. Statistical significance was determined using Student’s *t*-test: ****P < 0.0001, ***P < 0.001. (**B**) Cell illustration depicting pPHDD1-encoded genes that are part of the low-salt stimulon of *Pdd* RM-71. Cellular membrane was used from Servier Medical Art templates, licensed under a Creative Commons Attribution 3.0 Unported License. (**C**) Graph showing the distribution of upregulated (orange), downregulated (blue), and unchanged genes (gray) along pPHDD1 virulence plasmid of RM-71 at 1% NaCl versus 3% NaCl. All NCBI locus tags in *x*-axis must be preceded by “A0J47_RS.” “A” and “B” denote the groups where most of the upregulated genes are concentrated. (**D**) Heat map depicting upregulated and downregulated DEGs in RM-71 pPHDD1 plasmid at 1% NaCl versus 3% NaCl. Expression values are represented as Log_2_ (Fold Change); hypothetical proteins are denoted as “Hyp.”

Changes in expression at the transcriptional level may not necessarily reflect the final changes in actual protein amounts since other regulatory mechanisms may tune ultimate protein abundance (120). To gain an insight into the effect of salinity in the secretome of *Pdd*, RM-71 culture supernatants obtained at 3% NaCl and 1% NaCl were examined by SDS-PAGE, and protein bands were subjected to quantitative analysis. As shown in Fig. 9A, the majority of the secreted proteins were significantly more abundant at 1% NaCl than at 3% NaCl. Notably, cytotoxins Dly, PhlyP, and PhlyC and proteins of the putative PirAB-like toxin were more abundant in the profile at low salt. The most abundant protein bands corresponded to Dly and the PirA-like 11-kDa protein (A0J47_RS13280), which were 25 and 13 times more abundant, respectively, at 1% than at 3% NaCl (Fig. 9B). Altogether, these results clearly confirm that a shift from high to low salinity triggers a virulence profile in *Pdd*. In concordance with the results of the analysis of secreted proteins, the phenotypical plate tests for the detection of hemolysis (a phenotype attributable to synergistic effects of Dly with PhlyP and PhlyC) and phospholipase (a phenotype mainly attributable to Dly) showed increased activities at 1% compared to 3% NaCl (Fig. 10A). These observations are in the same line as previous reports documenting the induction of cytotoxin expression in *Vibrio* species in response to low salt, as seen with *V. vulnificus vvhA* hemolysin (121). Similarly, cytotoxicity of *V. parahaemolyticus* has been shown to be higher when grown in 1% NaCl when compared to 3% NaCl (107).

Additional secreted proteins with increased abundance at low salt were a lipoprotein (A0J47_RS11245) and an RstAB-dependent hypothetical protein (A0J47_RS20565). In accordance with these results, their respective genes were also upregulated at 1% NaCl in the transcriptomic assay. However, the trypsin-like serine protease (band 8 in Fig. 9A), which was 5.67-fold upregulated at 1% NaCl in the RNA-seq data, exhibited more protein abundance at 3% NaCl. It has to be noted that, due to the low amount of protein obtained from cultures grown to the exponential phase (OD_600_ of 0.55), the comparative analysis of the secretome at the two NaCl conditions was performed using cultures grown to the stationary phase (OD_600_ of 1.7). Therefore, it might be expected that the relative levels of some proteins in the *Pdd* secretome when comparing the two salinity conditions do not exactly mimic their relative levels in the transcriptomic data.

Altogether, our data showing that secreted proteins are much more abundantly produced at low salinity (Fig. 9) indicate that *Pdd* cells allocate valuable resources for the production of high amounts of proteins. Consistent with these observations, several genes involved in protein synthesis and regulation were transcriptionally induced at low salt (Fig. 2 and Table 2), and as many as 52 genes encoding ribosomal proteins were upregulated at 1% NaCl (Table S2). Additionally, the genes encoding ribosomal RpsF modification protein RimK (A0J47_RS00405) as well as YegQ (A0J47_RS12990), involved in tRNA hydroxylation, were listed among the 20 top DEGs under this condition.

### Iron acquisition systems are upregulated at 1% NaCl

Iron (Fe) is an essential element for most microorganisms, but its availability within the host is strongly limited, as it is chelated by proteins (122). In a vertebrate host, most Fe is complexed to heme, and hemoglobin is contained within erythrocytes. Previous studies reported that *Pdd* can use ferric citrate, ferrous iron, transferrin, and heme as iron sources (123, 124). Being low salinity an informative signal of entry into a host, it would be expected that iron uptake systems be upregulated at 1% in *Pdd*. Confirming this hypothesis, RNA-seq unveiled the upregulation, at low salt, of Fbp and Feo systems, involved in ferric and ferrous iron transport, respectively (Table 2). *fbpA* gene was the most upregulated with an FC of 18.74 (Table 2), but the upregulation was also observed for the gene encoding the TonB-dependent siderophore receptor FhuE and for some genes of the cluster encoding the heme iron utilization system (Fig. 3, chromosome I panel). Furthermore, the upregulation of erythrocyte-lysing hemolysins Dly and PhlyP at low salt is expected to contribute to the release of heme iron. Also of note, low salt conditions upregulated (9.5-fold) a plasmid (pPHDD1)-encoded TonB-dependent receptor (A0J47_RS20555) homologous (60% amino acid identity) to Vep20, a transferrin receptor that is a key element for *V. vulnificus* virulence in eels (125).

### Additional candidate virulence factors encoded within pPHDD1 plasmid are upregulated at low salt

Previous studies have demonstrated that the deletion of the pPHDD1-encoded cytotoxin genes *dly* and *hlyApl* in *Pdd* RM-71 is not sufficient to mimic the low-virulence profile of a naturally plasmidless strain of *Pdd* (44), emphasizing the potential implications of yet uncharacterized virulence factors encoded within pPHDD1 plasmid. As said above, the hemolytic and phospholipase activities are upregulated at low salinity and are mainly attributable to the cytotoxins encoded within pPHDD1 (Fig. 10A). By studying the comparative transcriptional landscape of pPHDD1 plasmid in response to low salt, we detected the upregulation of additional, potential virulence factors (Fig. 10B), whose genes are distributed in specific regions within the plasmid, suggesting that the genes within a cluster may be co-transcribed and co-regulated (Fig. 10C and D). A cluster A of five upregulated genes includes the AcrAB proteins, TolC, and two additional ABC permeases, where AcrAB-TolC constitutes a putative multidrug efflux transporter. Cluster B includes two interesting candidate virulence genes that have not been studied in *Pdd* so far. The first gene encodes an OM lipoprotein (A0J47_RS20550) homologous to Vep07 involved in resistance to eel serum in *V. vulnificus* biotype 2, and mutations in *vep07* were correlated with loss of virulence for eels (126). The second gene encodes the Vep20-like TonB-dependent transferrin receptor mentioned above (A0J47_RS20555). It is so far unknown whether these two genes encoding the Vep07- and the Vep20-like proteins play a role in *Pdd* pathobiology, but they certainly will deserve special attention in future studies. In addition, A0J47_RS20585, encoding an OM protein OmpU, is also part of the low salt-induced profile (FC 3.7). Several studies have disclosed the crucial role of OmpU in proliferation, colonization, and adhesion in different *Vibrios* (127). In accordance with our results, high salt also induced the upregulation of OmpU in *V. parahaemolyticus* (8). Finally, two uncharacterized pPHDD1-encoded proteins are also part of the low-salt stimulon of *Pdd* and correspond to gene A0J47_RS20580, induced 3.6-fold at 1% NaCl, and the T2SS-secreted RstAB-dependent protein (A0J47_RS20565; FC: 4.7) (27).

### Concluding remarks

Here, we propose that the decrease in NaCl concentration experienced by *Pdd* on entry into a host (from 3% to 1% NaCl) triggers a virulence program by strongly upregulating cytotoxin abundance in the secretome fraction, protein translation, expression of iron acquisition systems, and additional virulence-related functions. On the contrary, growth at marine salinity upregulates compatible solute uptake and enhances energy production mechanisms, utilization of trehalose and fructose as carbon sources, nitrogen metabolism, and global amino acid metabolism, with emphasis on enzymes of the ADS that might play a role in acid resistance. It is noticeable that the salinity regulon does not overlap with the previously reported RstAB regulon in *Pdd*, suggesting that this two-component system may not be primarily responsible for transducing information on changes in NaCl concentration. As an example, while mutation of RstAB system caused a 1,300-fold downregulation of capsule biogenesis genes (28), the impact of NaCl changes in the transcription of capsule genes in the present study was very discrete (with FCs ranging from 1.6 to 3.57). Among the species of the *Vibrionaceae*, few examples are known about the regulatory systems that transduce information on NaCl concentrations, but it is evident that transcriptome responses to salinity changes are transduced via several mechanisms in *Vibrios*, rather than via a single global regulator. In *V. cholerae*, OscR (6), CosR (7), and EnvZ/OmpR (128) constitute osmolarity-responsive regulators, and new regulatory genes have recently been described that mediate salinity-responsive modulation of gene expression in *V. parahaemolyticus* (129). The strong induction of a virulence genetic program in *Pdd* on exposure to low salinity, and the conspicuous changes elicited in the T2SS-dependent secretome, will surely boost future studies aimed at identifying novel osmolarity-sensitive systems in this generalist and highly versatile marine bacterium and will inspire studies in other *Vibrionaceae* species.

## Data Availability

A list of all differentially expressed genes (DEGs) is provided in Table S2. The complete genome of Pdd RM-71 strain is available under the accession number GCF_001708035.2.

## SUPPLEMENTAL MATERIAL

FIG S1 msystems.01253-22-s0001.tif

FIG S2 msystems.01253-22-s0002.tif

FIG S3 msystems.01253-22-s0003.tif

FIG S4 msystems.01253-22-s0004.tif

FIG S5 msystems.01253-22-s0005.tif

FIG S6 msystems.01253-22-s0006.tif

TABLE S1 msystems.01253-22-s0007.docx

TABLE S2 msystems.01253-22-s0008.xlsx

TABLE S3 msystems.01253-22-s0009.docx

ASM does not own the copyrights to Supplemental Material that may be linked to, or accessed through, an article. The authors have granted ASM a non-exclusive, world-wide license to publish the Supplemental Material files. Please contact the corresponding author directly for reuse.
