# Supplementary figures and images for: Low salinity activates a virulence program in the generalist marine pathogen Photobacterium damselae subsp. damselae
Source: mSystems. 2023 Jun 8;8(3):e01253-22. doi: 10.1128/msystems.01253-22 (PMC10308900; doi:10.1128/msystems.01253-22)

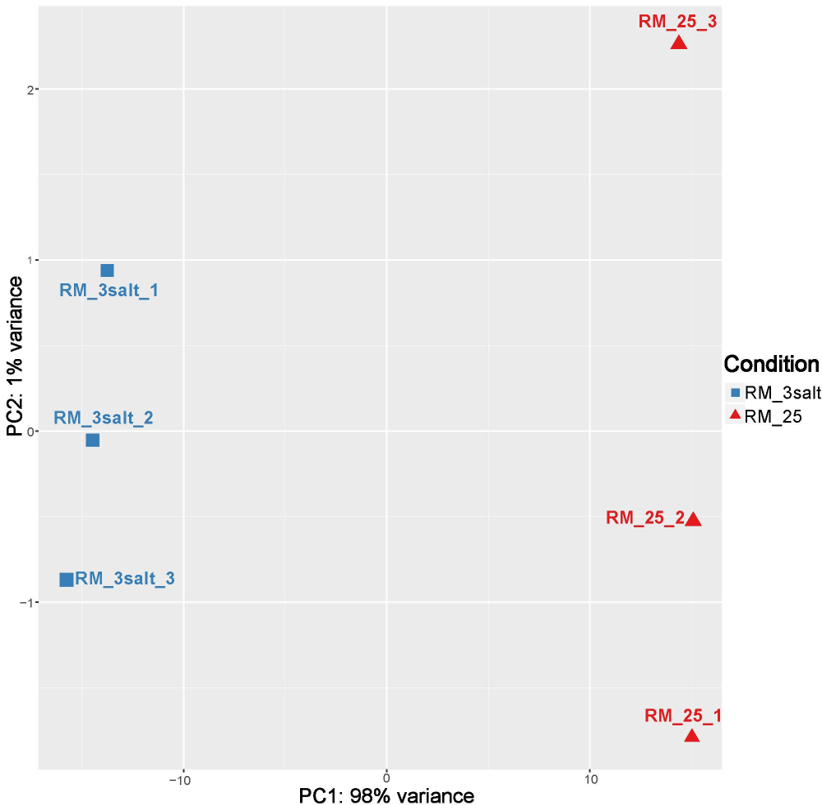

Supplement: FIG S1 [file msystems.01253-22-s0001.tif]

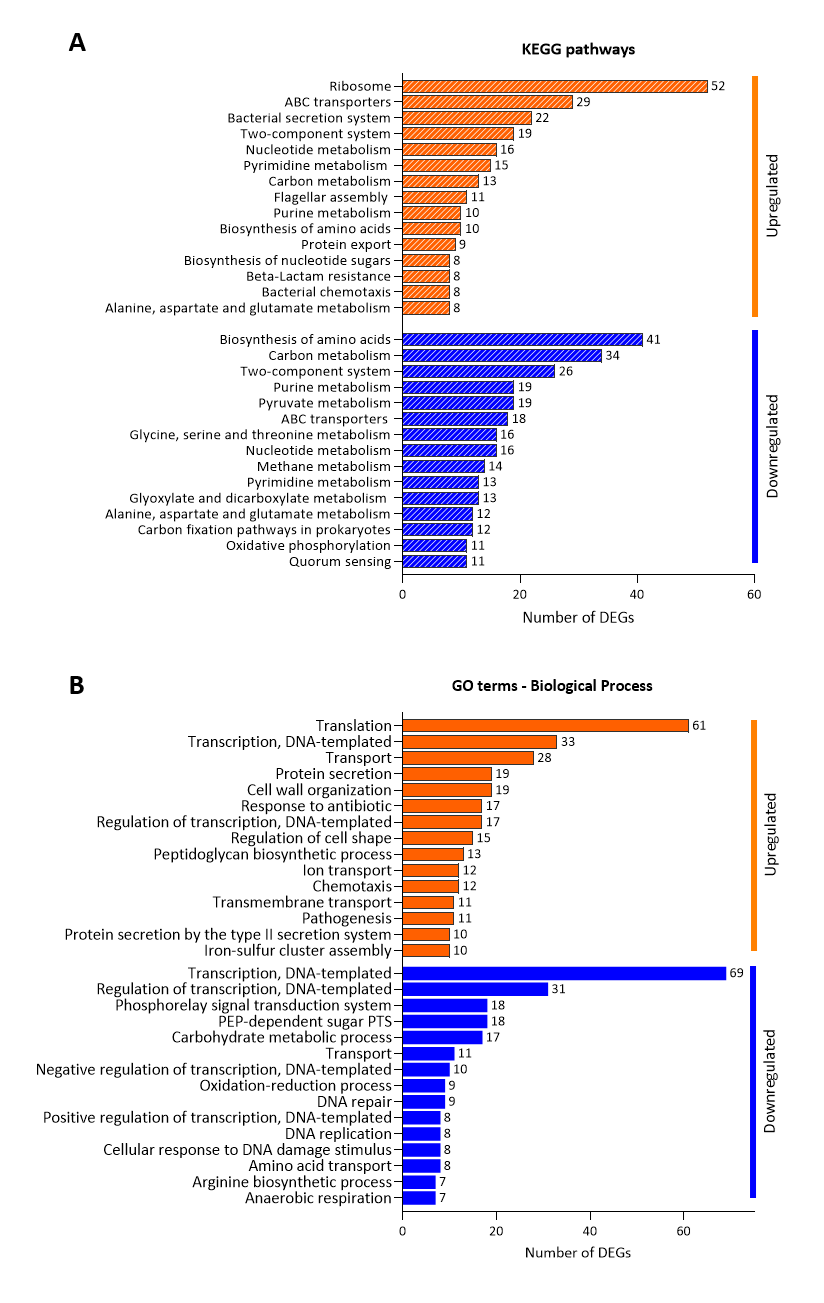

Supplement: FIG S2 [file msystems.01253-22-s0002.tif]

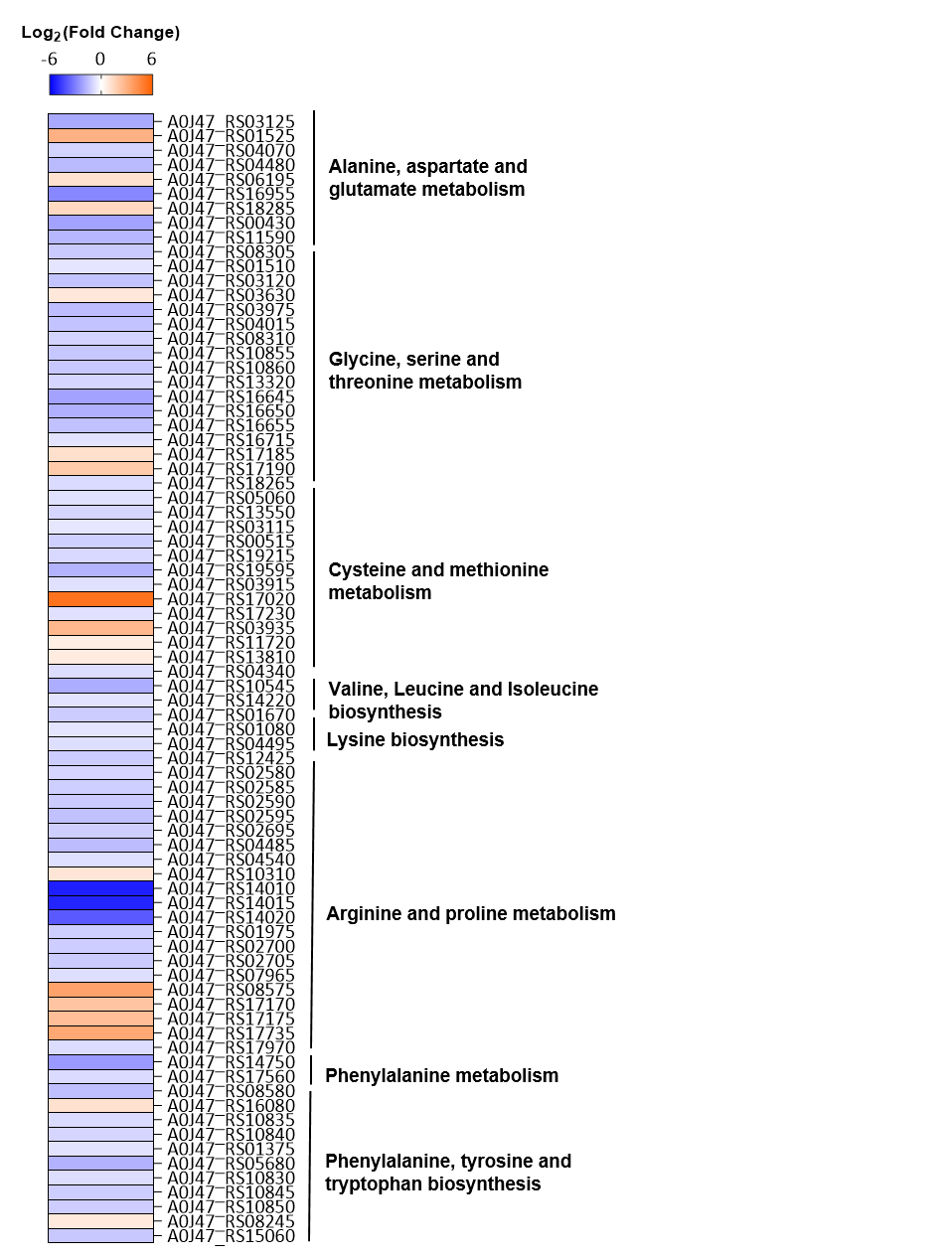

Supplement: FIG S4 [file msystems.01253-22-s0004.tif]

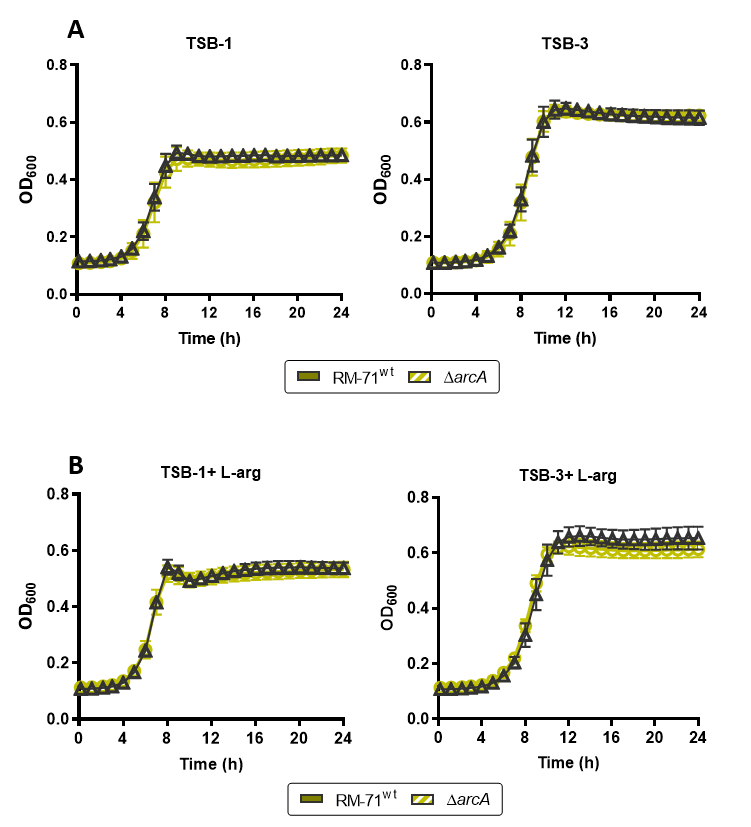

Supplement: FIG S5 [file msystems.01253-22-s0005.tif]

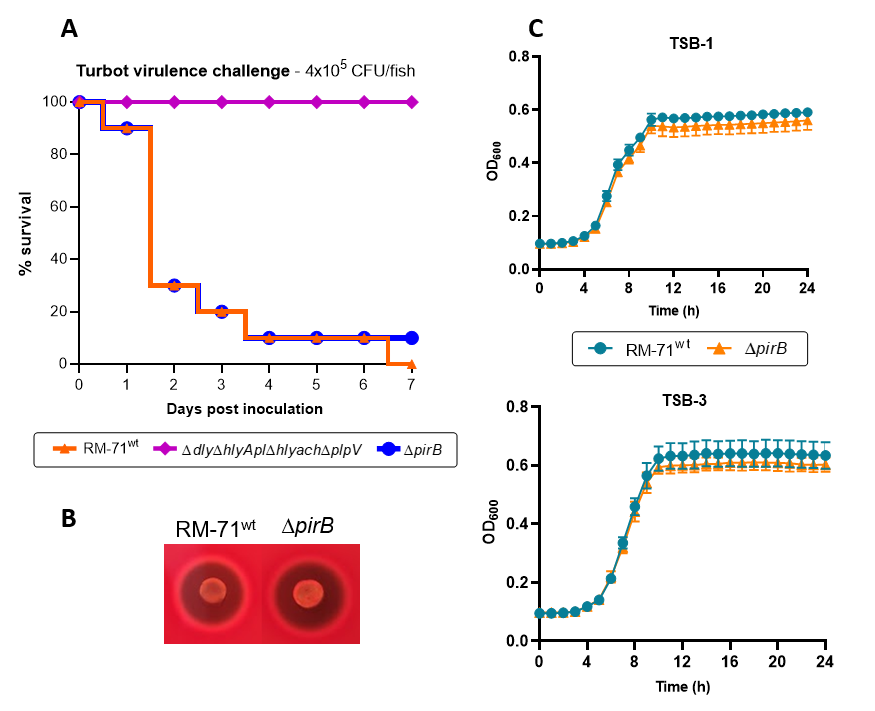

Supplement: FIG S6 [file msystems.01253-22-s0006.tif]
