# Supplementary material for: Low salinity activates a virulence program in the generalist marine pathogen Photobacterium damselae subsp. damselae
Source: mSystems. 2023 Jun 8;8(3):e01253-22. doi: 10.1128/msystems.01253-22 (PMC10308900; doi:10.1128/msystems.01253-22)
Supplement: TABLE S1 [file msystems.01253-22-s0007.docx]

| **Supplementary Table S1.** Detailed information about RNA sequencing results for each biological replicate | | | | |
| --- | --- | --- | --- | --- |
| **Sample** | **Replicate** | **Total reads** | **HQ Mapped reads** | **%HQ Mapped reads** |
| 1% NaCl | R1 | 58686274 | 57205008 | 97.48 |
|  | R2 | 62550784 | 61038519 | 97.58 |
|  | R3 | 55938626 | 54690361 | 97.77 |
| 3% NaCl | R1 | 47728092 | 46264033 | 96.93 |
|  | R2 | 46402608 | 45491032 | 98.04 |
|  | R3 | 54320954 | 53172666 | 97.89 |
