# Supplementary material for: Low salinity activates a virulence program in the generalist marine pathogen Photobacterium damselae subsp. damselae
Source: mSystems. 2023 Jun 8;8(3):e01253-22. doi: 10.1128/msystems.01253-22 (PMC10308900; doi:10.1128/msystems.01253-22)
Supplement: TABLE S3 [file msystems.01253-22-s0009.docx]

| **Supplementary Table S3.** Oligonucleotides used in this study | | | |
| --- | --- | --- | --- |
| **Target and name** | **Sequence (5’-3’)*** | **bq amplified** | **Reference** |
| ***pirB* (A0J47_RS13275) deletion** | | | |
| *1-2 fragment* | | | |
| Mut_*pirB*_1_BamHI | GC**GGATCC**GCAAAGTTCAAATCAAGAGT | 2020 | This study |
| Mut_*pirB*_2_SmaI | GC**CCCGGG**AGGTTTTTCTTGGGTTCCGC |  |  |
| *3-4 fragment* | | | |
| Mut_*pirB*_3_SmaI | GC**CCCGGG**TTCTCTTATTTCTTCCCAAA | 2011 | This study |
| Mut_*pirB*_4_XhoI | GC**CTCGAG**TCAGCGAGCTATTAGGTGTT |  |  |
| ***arcA* (A0J47_RS14010) deletion** | | | |
| *1-2 fragment* | | | |
| Mut_*arcA*_1_XhoI | GC**CTCGAG**gtcttcaccgtggaatgcag | 1998 | This study |
| Mut_*arcA*_2_PstI | GC**CTGCAG**cgctgcatgagctgcccaat |  |  |
| *3-4 fragment* | | | |
| Mut_*arcA*_3_PstI | GC**CTGCAG**acgatgaacaagaactcggc | 1985 | This study |
| Mut_*arcA*_4_NotI | GC**GCGGCCGC**gaagcggctgccattgacgt |  |  |
| ***pirB* screening** | | | |
| Mut_*pirB*_int_5’ | TCCATTTCGTTCTTGTTCGA | 370 | This study |
| Mut_*pirB*_int_3’ | GATTTTTCAACGGACGATAT |  |  |
| ***arcA* screening** | | | |
| Mut_*arcA*_int_5’ | gcgttaccgccaagtgcaac | 158 | This study |
| Mut_*arcA*_int_3’ | taagctggcatgatgctggc |  |  |
| ***Pdd* screening** |  |  |  |
| *ureD*_F | TCATACGTATTTACTGCATC | 396 | Osorio *et al*. (2000) |
| *ureD*_R | AGATAAAGTGATTCAAGAGA |  |  |
| * The underlined bold sequence denote the recognition sequence for restriction enzymes. | | | |
